# Nonpolysaccharide fraction of *Lonicerae japonicae* Flos attenuates cyclophosphamide-induced immunosuppression associated with modulation of the Keap1/Nrf2/HO-1/GPX4 signalling pathway

**DOI:** 10.3389/fphar.2026.1853492

**Published:** 2026-07-17

**Authors:** Mei Peng, Xiong Pan, Guanping Yao, Jie Zeng, Lang Zhou, Cui Hua, Ming Gao, Chengfeng Pan, Tingfei Deng, Liangqun Li, Xiaosheng Yang, Juan Yang

**Affiliations:** 1 State Key Laboratory of Discovery and Utilization of Functional Components in Traditional Chinese Medicine, School of Pharmaceutical Sciences, Natural Products Research Center of Guizhou Province, Guizhou Medical University Guiyang, Guiyang, China; 2 College of Pharmacy, Guizhou University of Traditional Chinese Medicine, Guiyang, China

**Keywords:** chlorogenic acid, cyclophosphamide, ferroptosis, immunosuppression, Keap1/Nrf2/HO-1/GPX4 pathway, *Lonicerae japonicae* Flos, natural products, non-polysaccharide fraction

## Abstract

**Background:**

Cyclophosphamide (CTX)-induced immunosuppression is closely associated with oxidative stress and ferroptosis, but effective therapeutic interventions remain limited. This study investigated the protective effects of the non-polysaccharide fraction of *Lonicerae japonicae* Flos (LJFE) against CTX-induced immunosuppression and explored its underlying molecular mechanism.

**Methods:**

LJFE was prepared by water extraction followed by ethanol precipitation, and its chemical profile was characterized using UPLC-MS/MS. Mice with CTX-induced immunosuppression were administered LJFE at doses of 100, 200, and 400 mg/kg for 10 consecutive days. Immune organ indices, serum IgE and IgM levels, oxidative stress markers (SOD, GSH, MDA), and the TBX21/GATA3 ratio were measured. The Keap1/Nrf2/HO-1/GPX4 signalling pathway was investigated via Western blotting and immunofluorescence. Serum metabolomics and molecular docking were applied to screen key differential metabolites and predict binding interactions between characteristic components and target proteins. LPS-stimulated RAW264.7 macrophages were used to verify pathway activation.

**Results:**

Seventeen chemical constituents were identified in LJFE. *In vivo*, LJFE markedly restored thymus and spleen indices, increased serum IgE and IgM levels by approximately 45% and 35%, respectively, elevated SOD and GSH activities, reduced MDA accumulation, and normalized the TBX21/GATA3 ratio. Mechanistically, LJFE downregulated Keap1 and upregulated Nrf2, HO-1, and GPX4 expression. Metabolomics analysis identified hydroferulic acid as a key differential biomarker involved in antioxidative regulation. Molecular docking suggested that characteristic components of LJFE exhibit favorable binding affinity toward Keap1 and Nrf2. *In vitro*, LJFE dose-dependently activated the Keap1/Nrf2/HO-1 signalling axis and suppressed excessive NO release in LPS-challenged macrophages.

**Conclusion:**

LJFE ameliorates CTX-induced immunosuppression by modulating the Keap1/Nrf2/HO-1/GPX4 pathway, thereby alleviating oxidative stress and ferroptosis. These findings support the potential of LJFE for further development as an immune-enhancing functional food ingredient.

## 1 Introduction

The immune system serves as the body’s core defense mechanism against pathogen invasion and maintains internal homeostasis; thus, its functional integrity is essential for human health (Chu et al., 2021). This system operates through the coordinated actions of immune organs (e.g., thymus and spleen), immune cells (e.g., T lymphocytes and macrophages), and immune molecules (e.g., antibodies and cytokines) to recognize and eliminate pathogens while surveilling abnormal cells. However, immune function is often compromised in clinical settings. For instance, chemotherapeutic agents such as cyclophosphamide (CTX)—a broad-spectrum antitumor and immunosuppressive drug—frequently induce nonspecific toxicity, resulting in thymic and splenic atrophy, T-cell subset imbalance, reduced antibody secretion, and subsequent secondary immunosuppression (Ding et al., 2014; Han C. et al., 2022; Zhang T. et al., 2022). Concurrently, CTX-induced excessive accumulation of reactive oxygen species (ROS) disrupts the body’s oxidative-antioxidative balance, triggering lipid peroxidation and activating ferroptosis—an iron-dependent form of programmed cell death—which further exacerbates immune cell damage and downregulates immune function (Chen et al., 2024; Mao et al., 2025). Current clinical interventions for CTX-induced immunosuppression predominantly rely on chemical immunoenhancers (e.g., lentinan). However, such agents have limitations including single-target actions and potential hepatorenal toxicity with long-term use (Ying et al., 2025). Therefore, developing safe, effective, and multi-target natural products has become an important research direction for ameliorating immunosuppressive states.

Natural products, especially medicinal plants with a history of ethnomedical use, exhibit unique advantages in immunomodulation due to their rich bioactive compounds and long-standing clinical practice (Alanazi et al., 2023). Honeysuckle (*Lonicerae japonicae* Flos, LJF), recorded in the Chinese Pharmacopoeia, has a long history of application in Traditional Chinese Medicine and various ethnic medical systems (e.g., Miao and Buyi medicine) (Shang et al., 2011). Traditionally used for clearing heat, detoxifying, and dispersing wind-heat, it is clinically employed for treating external febrile conditions, sore throat, and related symptoms, with its efficacy closely associated with anti-inflammatory, antibacterial, and immunomodulatory activities (Alanazi et al., 2023; Orsavová et al., 2022). Modern pharmacological studies further confirm that the diverse physiological activities of LJF stem from its abundant active constituents, including phenolic acids (e.g., chlorogenic acid, caffeic acid), flavonoids (e.g., quercetin, luteolin), and polysaccharides (Pham et al., 2022; Xiong et al., 2022). Among these, LJF polysaccharides have been shown to ameliorate CTX-induced immunosuppression in mice by enhancing macrophage phagocytosis and upregulating antibody levels (Zhang T. et al., 2022). However, the non-polysaccharide fraction of the LJF aqueous extract—which is rich in phenolic acids and flavonoids such as chlorogenic acid, luteolin, and quercetin—has received little attention. Whether this non-polysaccharide fraction (hereinafter referred to as LJFE) possesses immunomodulatory activity and, if so, whether it acts through the Keap1/Nrf2/HO-1/GPX4 pathway to counteract CTX-induced immunosuppression and ferroptosis remain unknown.

Therefore, the present study focuses specifically on LJFE, aiming to elucidate its immunomodulatory effects and underlying molecular mechanisms using a CTX-induced immunosuppressed mouse model and LPS-stimulated RAW264.7 macrophages.

Notably, phenolic acids (e.g., chlorogenic acid) and flavonoids, as quality control markers for LJF (Chinese Pharmacopoeia, 2020 Edition, Part I) (Xiong et al., 2022; Zhang et al., 2024), have been demonstrated to possess significant antioxidant activity—alleviating oxidative stress damage by scavenging ROS and activating antioxidant signalling pathways (Ge et al., 2023; Lai et al., 2022). Recent studies have revealed that dysregulation of the Keap1/Nrf2/HO-1/GPX4 signalling pathway, mediated by oxidative stress, is a core mechanism in CTX-induced immunosuppression and ferroptosis: Keap1 (Kelch-like ECH-associated protein 1) binds to Nrf2 (nuclear factor erythroid 2-related factor 2) to promote its ubiquitin-dependent degradation. Nrf2, as a master transcriptional regulator of antioxidant response, upon activation, regulates the expression of downstream HO-1 (heme oxygenase-1) and GPX4 (glutathione peroxidase 4). HO-1 reduces ROS production by degrading heme, while GPX4 directly antagonizes ferroptosis by inhibiting lipid peroxidation (Zhu et al., 2025).

To achieve these objectives, first, the major chemical constituents of LJFE were identified using ultra-performance liquid chromatography-tandem mass spectrometry (UPLC-MS/MS). Subsequently, the restorative effects of LJFE on immune function were evaluated through immune organ indices, serum antibody levels (IgE, IgM), oxidative stress markers (SOD, GSH, MDA), and T lymphocyte subset balance (TBX21/GATA3 ratio). Furthermore, combining Western blotting, immunofluorescence, and metabolomics, the study explores the molecular mechanism by which LJFE regulates oxidative stress and ferroptosis via the Keap1/Nrf2/HO-1/GPX4 pathway, and predicts the interactions between key compounds (e.g., chlorogenic acid) and pathway proteins through molecular docking. This work aims to provide a scientific basis for the application of *L. japonica* Flos in the food industry and to offer new insights into the development of functional food products with immune-regulating properties.

## 2 Materials and methods

### 2.1 Preparation of the nonpolysaccharide fraction from LJF

The sample was collected from Chicun village, Yuxi town, Daozhen County, Zunyi city (longitude: 107.63993279736°, latitude: 28.8820117241°). The sample was identified as the flower of *Lonicera japonica* Thunb (*Lonicerae japonicae* Flos) by Yunfei Tan from the Natural Product Research Center of Guizhou Province. The voucher specimen (No. 2023-JYH-01) is deposited at the Natural Products Research Center of Guizhou Province. The dried and finely ground LJF (1000 g) was extracted with deionized water (3 × 10 L, for 2 h each) under reflux, followed by concentration under reduced pressure. Six times the amount of 95% ethanol was added to the extract to precipitate the polysaccharides, which were then filtered. The ethanol fraction was collected and concentrated under reduced pressure until it became an extract (LJFE). The prepared LJFE was numbered as Batch No. LJFE-20230901 and further dried by vacuum freeze-drying. The yield of LJFE was 8.25% (w/w) from dried starting material. All LJFE samples were sealed and stored at −20 °C in the dark. Three independent batches of LJFE were prepared in parallel in this study, and the yield of LJFE had a relative standard deviation (RSD) of less than 5%, indicating good batch-to-batch consistency.

### 2.2 Component identification of LJFE

An appropriate amount of LJFE was dissolved in methanol and filtered. The sample was then injected under the following chromatographic conditions: Hypersil GOLD with a Q column (100 mm × 2.1 mm, 1.9 μm), with a mobile phase consisting of acetonitrile (A) and 0.1% methanol in water (B) for gradient elution, at a flow rate of 0.3 mL/min and a column temperature of 40 °C. The mass spectrometry conditions were set as follows: positive ion mode was used for the Orbitrap high-resolution mass spectrometer, with a spray voltage of 3 kV, auxiliary gas at 10 arb, sheath gas at 35 arb, and an ion transfer tube temperature of 320 °C. The chemical compounds of LJFE were initially identified via UPLC‒MS/MS (QE Focus, Thermo Fisher Scientific).

### 2.3 Animals

Sixty BALB/c mice (female; weight, 16–18 g; age, 6–8 weeks) were provided by Beijing SPF Biotechnology Co. Ltd. (Beijing, China). The mice were acclimatized for 1 week before the experiment and maintained at a temperature of 22 °C ± 2 °C and humidity of 40%–60% with free access to drinking‐water and natural light. This study was reviewed and approved by the Experimental Animal Ethics Committee of Guizhou Medical University (SYXK: GUI 2023-0002).

### 2.4 Establishment and grouping of the immunosuppressed mouse model

After 1 week of acclimatization, 60 BALB/c mice were randomly divided into the control, model (cyclophosphamide, CTX), positive control (lentinan tablets, positive), low-dose (LJFE-L), middle-dose (LJFE-M) and high-dose (LJFE-H) LJFE groups, with 10 mice in each group. Lentinan tablets (GuoYaoZhunZi H42022609, Wuhan Di Ao Pharmaceutical Co., Ltd.) and cyclophosphamide (GuoYaoZhunZi HJ20160467, Baxter Oncology GmbH, Germany) were used. CTX was prepared as a 7 mg/mL solution in control saline at a dose of 70 mg/kg body weight, as previously described (Ding et al., 2014; Zhao et al., 2024; Li et al., 2022). Except for those in the control group, the mice in the other groups were injected with the prepared CTX solution at 0.1 mL/10 g body weight for 5 d, followed by gastric gavage. The control and model groups were administered control saline by gavage, whereas the positive control group received 45 mg/kg body weight lentinan solution. LJFE doses were selected based on FDA guidance using body surface area conversion (km coefficient). According to the *Chinese Pharmacopoeia* (2025 edition), the clinical dose of *Lonicerae japonicae* Flos is 6–15 g, corresponding to a mouse equivalent crude drug dose of 3.0 g/kg. With an LJFE extraction yield of 8.25%, the crude drug equivalent was 250 mg/kg. Based on preliminary experiments, 100, 200, and 400 mg/kg were chosen as the low, medium, and high doses, respectively.

### 2.5 Sampling and detection

After 10 d, blood was collected from the mice through the angular vein 1 h after administration. The serum was separated via centrifugation at 3500 r/min and stored at −80 °C. A portion of the serum was used to measure the IgE, IgM, and SOD activity; GSH and MDA levels. The remaining serum was used for blood metabolomic analysis. At the end of the experiment, the mice were euthanized, and their thymus and spleens were collected, weighed, and washed with PBS. The thymus and a portion of the spleens were fixed in 4% paraformaldehyde (PFA) for HE histological sectioning, and a portion of the spleen tissue was stored at −80 °C for proteomics analysis.

### 2.6 Measurement of thymus and spleen indices

The mice were weighed and recorded prior to the experiment. The thymus/spleen index (mg/g) was calculated via the following formula:

$$\text{thymus}/\text{spleen index}=\frac{\text{thymus}\;\left(\text{spleen}\right)\text{mg}\;}{\text{body}\;\text{weight}\;\left(g\right)}\times 10$$

### 2.7 Biochemical indicator measurements

Mouse IgE (J2058-A, 202303, Cation, China), IgM (J2057-A, 202303, Cation, China), and SOD, GSH, and MDA biochemistry kits (J2698-A, 202303, Cation, China) were used to measure the levels of the immune proteins IgE and IgM and the oxidative stress-related enzymes SOD, GSH and MDA in the serum. All procedures were performed according to the manufacturer’s instructions.

### 2.8 Histopathology examination

Mice thymus and spleen tissues were fixed in 4%–10% neutral formaldehyde, embedded, fixed, sectioned, and stained with haematoxylin and eosin (HE). The sections were observed under an optical microscope (BX5; Olympus, Tokyo, Japan) and photographed via a digital camera (MSX2-H; Mshot, Guangzhou, China). Inflammatory tissue sections were examined to assess the damage caused by CTX in the mouse thymus and spleen cells.

### 2.9 Serum metabolomics

Thawed and centrifuged serum was extracted using a 20% acetonitrile/methanol internal standard. After centrifugation at 12,000 r/min, the supernatant was transferred to a refrigerator and stored at −20 °C until it was allowed to settle. Next, the supernatant was centrifuged again at 12,000 r/min for 3 min at 4 °C. Finally, 180 μL of the supernatant was transferred to the insert of the corresponding sample vial for instrumental analysis. The data acquisition instrument system consisted primarily of a UPLC (ExionLC AD, https://sciex.com.cn/) and a quadrupole time‒of‒flight mass spectrometer (Q-TOF-MS/MS, TripleTOF 6600, AB SCIEX). Through systematic detection and analysis, the metabolism of the different compounds of LJFE in mice and disease-related metabolic pathways were determined.

### 2.10 Immunofluorescence histology

Ten days after the experiment, paraffin sections of the spleen were dewaxed with water for antigen retrieval. After washing three times with PBS, the primary antibody was added, followed by incubation with the second primary antibody. Next, the secondary antibody was added for incubation and washing, and 4′,6-diamidino-2-phenylindole (DAPI) was used to counterstain the cell nuclei. After incubation, the sections were sealed with anti-fluorescence quenching mounting medium and observed under an optical microscope for data collection and photography.

### 2.11 Western blot analysis of spleen tissue

Western blotting was used to detect the expression levels of Nrf2, Keap1, HO-1, and GPX4 in mouse spleen tissue homogenates from the *in vivo* experiment. Total protein was extracted from spleen tissue using a protein extraction kit (Servicebio, G2026). After quantification, proteins were separated by SDS-PAGE, transferred to PVDF membranes, and blocked with 5% skim milk. Primary antibodies against Nrf2 (CST #12721, 1:1000), Keap1 (Boster, A00514-3, 1:1000), HO-1 (Proteintech, 81281-1-RR, 1:1000), GPX4 (Boster, BM5231, 1:1000), and β-actin (Servicebio, GB12001, 1:5000) were added and incubated overnight at 4 °C. After washing, HRP-conjugated secondary antibody (1:5000) was applied. Signals were detected by enhanced chemiluminescence (ECL) and visualized using a gel imaging system. β-actin served as the loading control.

### 2.12 Molecular docking

The crystal structures of the proteins with anti-inflammatory activity, PDB ID: Keap1 (4IFJ), Nrf2 (8IVR), HO-1 (1IRM), and GPX4 (8Q8J), with a resolution of 3.80 Å were downloaded from the RCSB PDB (https://www.rcsb.org/). For molecular docking, AutoDock 4 software was used, and all proteins and ligands were prepared via the AutoDockTools-1.5.6 wizard. Initially, proteins were prepared by removing water molecules and heteroatoms, adding missing amino acids and polar hydrogens, applying Kollman charges, assigning AD4 atom types and saving them in the PDBQT receptor file. Simultaneously, the energy of the ligands was minimized by the default force field in Avogadro to optimize their geometries and was saved in pdb format. Furthermore, the ligand molecules were docked into the receptor binding site via molecular docking software to predict their binding affinity and interaction patterns.

### 2.13 Cell experiments (RAW264.7 cells)

RAW264.7 cells were purchased from Keycell Biotechnology (Wuhan, China). The primary antibodies against Keap1 (Boster, A00514-3), Nrf2 (Proteintech, 80593-1-RR), HO-1 (Proteintech, 81281-1-RR), GPX4 (Boster, BM5231) and β-actin (Servicebio, GB12001) were used in this experiment. Lipopolysaccharide (LPS, Solarbio, L8880), Dexamethasone (DEX), Cell Counting Kit-8 (CCK-8, Solarbio, CA1210) and Nitric oxide (NO) assay kit (Beyotime, S0021S) were applied for subsequent detection. Dulbecco’s Modified Eagle Medium (DMEM, Thermo), fetal bovine serum (FBS, Thermo) and penicillin/streptomycin (Beyotime) were used for cell culture.

RAW264.7 cells were cultured in DMEM medium containing 10% (v/v) FBS and 1% (v/v) penicillin/streptomycin at 37 °C in a humidified incubator with 5% CO_2_. The medium was replaced every 48 h, and cell passage was performed every 48-72 h.

For cell viability analysis, logarithmic growth phase RAW264.7 cells were seeded in 96-well plates at a density of 3 × 10^3^ cells/well and incubated for 24 h for adherence. The cells were treated with LJFE at different concentrations (2, 4, 6, 8, 12, 16, 20 mg/mL), in the presence of LPS (500 ng/mL), with a blank control group set. After 24 h of incubation, 10 μL of CCK-8 reagent was added to each well and incubated for an additional 2 h, and the absorbance was measured at 450 nm to evaluate the cytotoxicity of LJFE. The LPS concentration (500 ng/mL) was chosen based on literature (Lin et al., 2025; Wenju et al., 2022) and pilot experiments as an optimal dose for inducing inflammation without excessive cytotoxicity.

For the determination of NO secretion, RAW264.7 cells in logarithmic growth phase were seeded in 24-well plates at 1 × 10^5^ cells/well, and the experiment was divided into 6 groups: blank control group, LPS group, LPS+DEX group (positive control), LPS+LJFE-L (4 mg/mL) group, LPS+LJFE-M (8 mg/mL) group, and LPS+LJFE-H (16 mg/mL) group. After 24 h of cell seeding, LPS and corresponding concentrations of LJFE were added, and the cells were incubated for another 24 h. The supernatant was collected, and the NO level was determined using the NO assay kit according to the manufacturer’s instructions. Endotoxin contamination was minimized by preparing LJFE under strict aseptic conditions and filtering through a 0.22 μm membrane filter. A blank control and solvent control were included to exclude non-specific interference.

Western blot analysis for RAW264.7 cells: After treatment, RAW264.7 cells were washed twice with PBS and lysed in RIPA buffer (Servicebio, G2002) containing protease inhibitors (PMSF). Total protein concentration was quantified using a BCA kit (Beyotime, P0012). Equal amounts of protein (30 μg per lane) were separated by 10% SDS-PAGE and then transferred to PVDF membranes (Millipore, ISEQ00010). The membranes were blocked with 5% non-fat skim milk in TBST for 1 h at room temperature, followed by overnight incubation at 4 °C with the same primary antibodies (anti-Keap1, anti-Nrf2, anti-HO-1, anti-GPX4, and anti-β-actin) as described in Section 2.11. After washing with TBST, the membranes were incubated with HRP-conjugated secondary antibody (1:5000, Servicebio, GB23303) for 1 h at room temperature. Protein bands were visualized using an enhanced chemiluminescence (ECL) kit (Servicebio, G2014) and captured with a gel imaging system (Tanon 5200). Band intensities were quantified using ImageJ software, and β-actin was used as the loading control.

### 2.14 Data processing and analysis

Data analysis was performed via SPSS statistical software (version 22.0; SPSS Inc). Statistical differences among multiple groups were analysed via one-way analysis of variance (ANOVA), followed by Tukey’s test for multiple mean comparisons. All numerical data are expressed as the mean ± standard deviation (SD), and statistical significance was set at P < 0.05. Graphs were created via Prism 8.0.2.

## 3 Results

### 3.1 Chemical composition analysis of nonpolysaccharide fraction of Lonicerae japonicae flos

**FIGURE 1:**
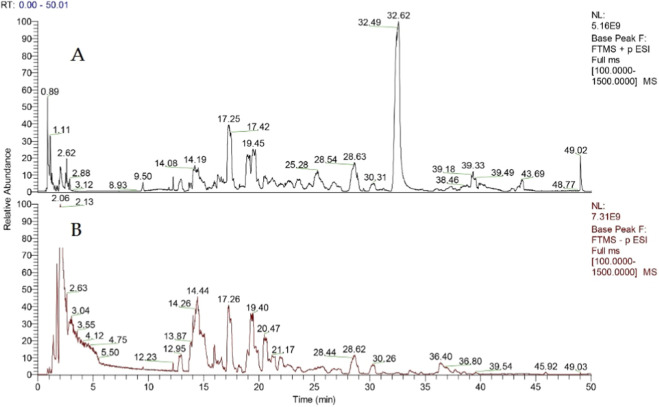
Ion chromatograms of the nonpolysaccharide fraction of Lonicerae japonicae Flos in positive and negative ion modes (**(A)** ESI−, **(B)** ESI+).

By utilizing a literature-based chemical database of LJF and guided by retention times, accurate molecular weights, and secondary fragment ion information, the compounds of the Nonpolysaccharide Fraction of LJF were annotated in this study, as detailed in Table 1. The Base Peak Chromatogram (BPC) of the Nonpolysaccharide Fraction of *Lonicerae japonicae* Flos is presented in Figure 1. A total of 17 chemical compounds were identified, including 10 flavonoids, 5 organic acids, 1 coumarin, and 1 phthalate ester. Key compounds included chlorogenic acid (quality control marker of LJF), quercetin, and luteolin (immunomodulatory flavonoids), which is consistent with previous reports on the chemical composition of LJF (Cai et al., 2019; Senwang et al., 2022).

**TABLE 1:** Analysis of ingredients in LJFE.

No.	Compound	RT/min	Theoretical m/z	Observed m/z	Mass error	Adduction	MS/MS	Compound Class
1	Chlorogenic acid	2.643	354.31	354.102	−0.208	[M+H]+	355.24374, 163.03827, 135.04355	Organic acids
2	Quinate	2.648	192.17	192.056	−0.114	[M-H]-	191.05547, 59.01226, 155.03300	Organic acids
3	Catechin	9.251	290.27	290.086	−0.192	[M+H]+	291.08453, 123.04373, 139.03842	Flavonoids
4	Esculetin	9.706	178.14	178.019	−0.121	[M+H]+	177.01775, 133.02870, 89.03799	Coumarins
5	Caffeic acid	9.928	180.16	180.035	−0.125	[M-H]-	179.03336, 135.04346, 133.02765	Organic acids
6	Epicatechin	11.18	290.27	290.072	−0.198	[M-H]-	289.07050, 109.02781, 137.02281	Flavonoids
7	Astragalin	13.19	448.38	448.108	−0.272	[M+H]+	449.17578, 287.05377, 85.02855	Flavonoids
8	Azelaic acid	14.4	188.22	188.097	−0.123	[M-H]-	187.09590, 169.08549, 97.06415	Organic acids
9	3,5-Dicaffeoylquinic acid	14.707	516.45	516.136	−0.314	[M+H]+	163.03821, 135.04347, 117.03321	Organic acids
10	2-(3,4-Dihydroxyphenyl)-6,7-dihydroxychroman-4-one	16.94	288.25	288.056	−0.194	[M-H]-	287.05478, 135.04343, 151.00194	Flavonoids
11	Quercetin	17.268	302.24	302.539	0.299	[2M-H]-	301.03394, 65.00167, 121.02785	Flavonoids
12	Luteolin	17.29	286.24	286.04	−0.200	[M-H]-	285.03915, 133.02777, 107.01212	Flavonoids
13	Tiliroside	17.581	594.52	594.145	−0.375	[M+H]+	309.09613, 287.05362, 119.04884	Flavonoids
14	Phloretin	18.84	274.27	274.077	−0.193	[M-H]-	273.07568, 93.03287, 65.00165	Flavonoids
15	Apigenin	18.971	270.24	270.046	−0.194	[M-H]-	271.08590, 153.01753, 119.48881	Flavonoids
16	Diosmetin	32.14	300.26	300.071	−0.189	[M+H]+	301.13962, 258.05129, 286.04507	Flavonoids
17	Dehp	43.696	390.56	390.284	−0.276	[M+H]+	391.24313, 167.00021, 71.08600	Phthalate esters

### 3.2 Effects of LJFE on immune organ indices in mice

**FIGURE 2:**
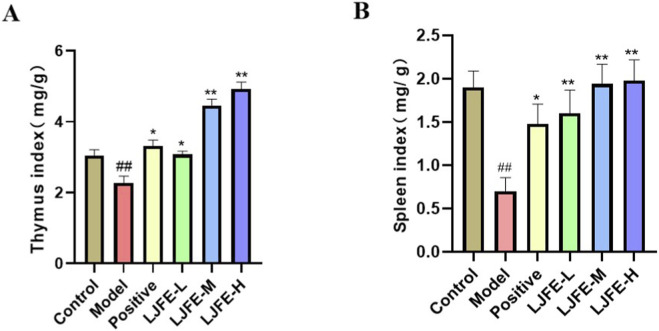
Thymus and spleen indices in immunosuppressed mice after LJFE administration. **(A)** Thymus index; **(B)** Spleen index. Data are mean ± SD (n = 10). #P < 0.05, ##P < 0.01 vs. control group; *P < 0.05, **P < 0.01 vs. model group.

**FIGURE 3:**
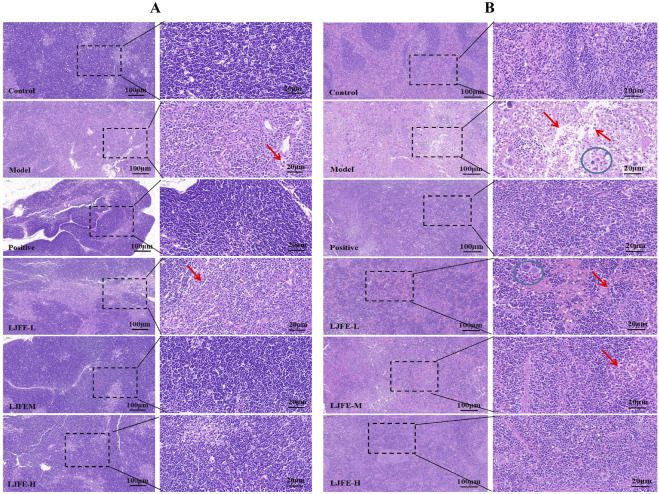
Histopathological changes of thymus and spleen in CTX-treated mice (HE staining, 100× and 400×). **(A)** Thymus sections; **(B)** Spleen sections. Control: Normal tissue structure; Model: Severe cell damage; LJFE-L: LJFE low dose; LJFE-M: LJFE medium dose; LJFE-H: LJFE high dose.

As shown in Figure 2, LJFE significantly increased the organ indices of immunosuppressed mice compared with those of model mice (P < 0.05 or P < 0.01). HE-stained histological sections revealed that in the control group (Figure 3), the thymus and spleen cells were densely and orderly arranged, with clear nuclei and distinct boundaries between the red and white pulp. In contrast, the model group exhibited a sparse and disordered cell arrangement with solidified nuclei and indistinct boundaries between the red and white pulp. However, after treatment with LJFE, LJFE-L group showed mild improvement (reduced nuclear pyknosis); the LJFE-M and LJFE-H groups presented tightly arranged thymus and spleen cells with distinct boundaries and clear nuclei, resembling those in the control group.

### 3.3 Effects of LJFE on immune-related biochemical parameters in mice serum

**FIGURE 4:**
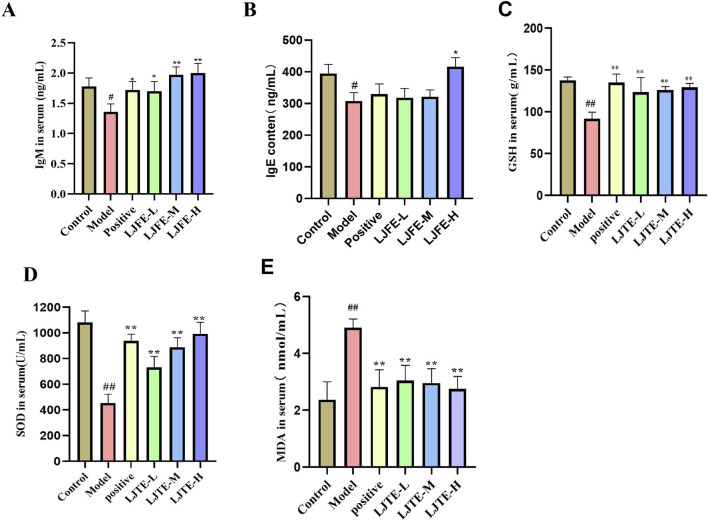
Effects of LJFE on immune-related biochemical parameters in the serum of immunosuppressed mice. **(A)** The level of IgM in serum. **(B)** The level of IgE in serum. **(C)** The levels of GSH in serum. **(D)** The activity of SOD in the serum. **(E)** The levels of MDA in serum. Compared with the control group, #P < 0.05, ##P < 0.01; compared with the model group, *P < 0.05, **P < 0.01.

As shown in Figure 4, after CTX-induced immunosuppression, bone marrow suppression and B lymphocyte damage occurred, resulting in significantly lower serum IgE and IgM levels in mice compared with the control group (P < 0.05). Following LJFE administration by gavage, both IgE and IgM levels improved. Compared with the model group, the IgE levels in the LJFE-M and LJFE-H groups were significantly higher (P < 0.01). In addition, the IgM level in the LJFE-H group was restored to the control level. After modeling, due to CTX-induced oxidative stress, the SOD activity in the model group was significantly lower than that in the control group, while all LJFE groups showed increased SOD activity compared with the model group (P < 0.01), all returning to normal levels. Meanwhile, GSH levels in all three LJFE groups were significantly higher than those in the model group, with the high dose approaching normal levels. Conversely, the MDA level in the model group was significantly higher than that in the control group, but after LJFE gavage, MDA levels in all treatment groups were significantly reduced compared with the model group (P < 0.01).

### 3.4 Effects of LJFE on the serum metabolomics of mice

#### 3.4.1 Principal component analysis (PCA)

**FIGURE 5:**
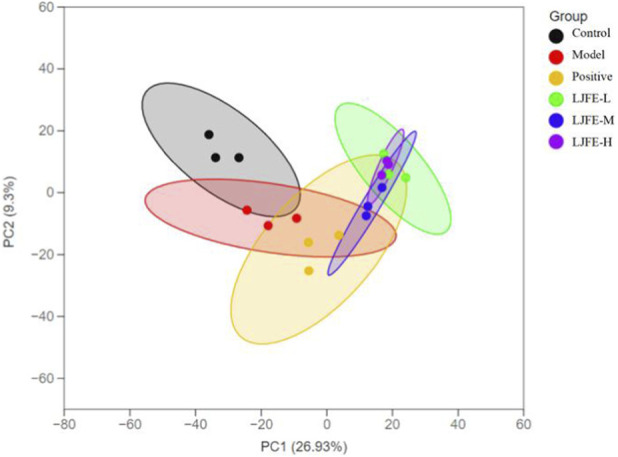
PCA plot of serum metabolomics for each group.

Raw data acquired from Q-TOF-MS/MS were preprocessed, and principal component analysis (PCA) was subsequently performed on the normalized peak area data matrix, with the results presented in Figure 5. PC1 and PC2 accounted for 26.93% and 9.3% of the total metabolic variance, respectively, and all mouse serum samples in each group were distributed within the 95% confidence interval, indicating good homogeneity and low individual differences within each group. Notably, a distinct intergroup separation was observed in the PCA 2D score plot, with no overlapping of metabolic profiles among the groups.

#### 3.4.2 Screening of differentially abundant metabolites and analysis

**FIGURE 6:**
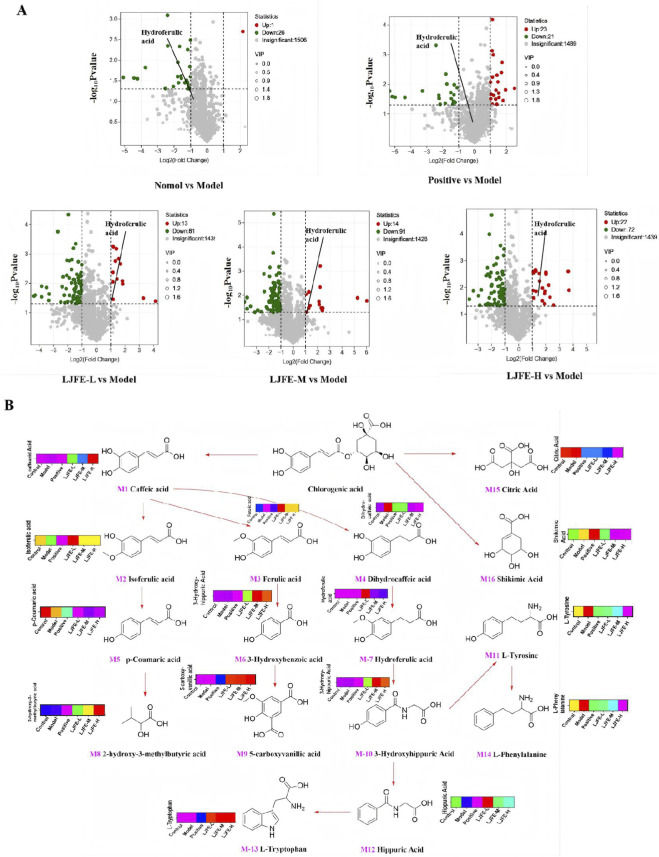
The volcano plot of hydroferulic acid among different groups of immunocom-promised mice and the possible metabolic pathways. **(A)** Differential metabolic volcano plot of hydroferulic acid among different groups of immunosuppressed mice. **(B)** Possible metabolic pathways of chlorogenic acid in mice.

To further identify differentially abundant metabolites, serum differential metabolites were screened using the OPLS-DA model (VIP >1) combined with t-test (P < 0.05). A total of 174 and 94 significantly altered metabolites were identified in the LJFE vs. control and LJFE vs. model comparisons, respectively (Supplementary Material 1). To identify potential biomarkers, univariate ROC curve analysis was performed using MetaboAnalyst 5.0 with a threshold of AUC >0.8. Hydroferulic acid was markedly upregulated following LJFE treatment, with an AUC exceeding 0.8 (Figure 6A) (Supplementary Material 1).

Notably, the MS response intensity of hydroferulic acid was significantly higher in all LJFE-treated groups compared to the model, control, and positive control groups. Consistent with previous reports (Liu et al., 2024), hydroferulic acid is a major *in vivo* metabolite of chlorogenic acid. Tracing the metabolic pathway of chlorogenic acid revealed multiple secondary metabolites derived from it in serum (see Supplementary Material). The volcano plot (Figure 6A) further confirmed the pronounced upregulation of hydroferulic acid across all LJFE dose groups.

To comprehensively characterize chlorogenic acid-related metabolic changes, systematic comparison and cluster analysis were performed, generating a heatmap of these metabolites (Figure 6B). Sixteen secondary metabolites showed significant alterations in the LJFE groups, clearly distinguishing them from the blank and model groups.

**FIGURE 7:**
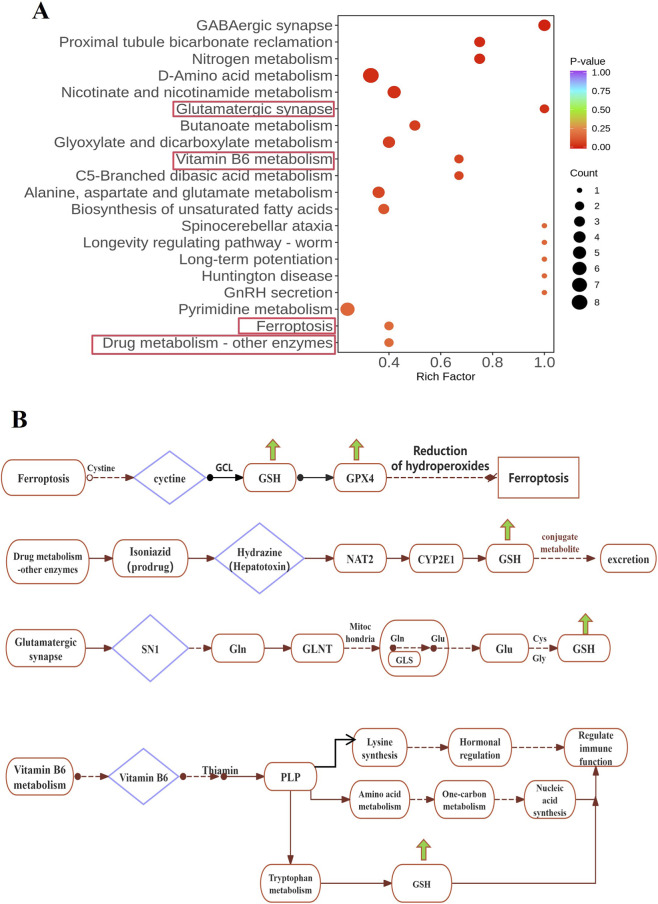
The immune-related diseases and metabolic pathways enriched by KEGG. **(A)** Relevant pathways enriched by KEGG. **(B)** Metabolic pathways of the pathways related to oxidative stress and immune regulation.

To explore the biological functions of these differential metabolites, KEGG pathway enrichment analysis was conducted. Twenty disease-related metabolic pathways were significantly enriched (Figure 7A), among which four were closely associated with anti-oxidative stress and immune regulation (Figure 7B): Glutamatergic synapse (Supplementary Material 2: https://www.genome.jp/dbget-bin/www_bget? map04724), Vitamin B6 metabolism (Supporting Material elsx2: https://www.genome.jp/dbget-bin/www_bget? map04977), Drug metabolism—other enzymes (Supplementary Material 2: https://www.genome.jp/dbget-bin/www_bget? map00983), and Ferroptosis (Supplementary Material 2: https://www.genome.jp/dbget-bin/www_bget? map04216).

### 3.5 Immunofluorescence detection

**FIGURE 8:**
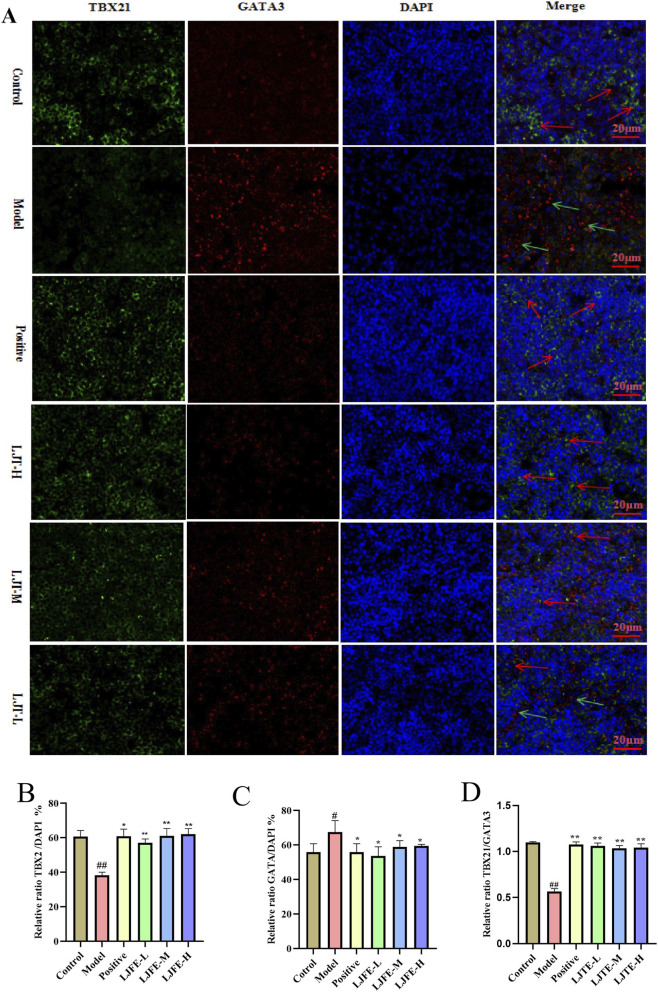
Effects of LJFE on the immunofluorescence expression of the TBX21 and GATA3 proteins in T cells and the positive expression rate. **(A)** Immunofluorescence expression of the TBX21 and GATA3 proteins (400×); **(B,C)** Positive expression rates of the TBX21 and GATA3 proteins. **(D)** Relative ratio of TBX21/GATA3. Note: The red arrow represents GATA3, and the green arrow represents TBX2. Compared with the control group, #P < 0.05, ##P < 0.01; compared with the model group, *P < 0.05, **P < 0.01.

As shown in Figure 8, compared with that in the control group, the expression of TBX21 was downregulated, whereas the expression of GATA3 protein was upregulated, with a significant difference in model group (P < 0.01, P < 0.05). The ratio of TBX21 to GATA3 was severely imbalanced. After treatment with LJFE, the expression of both the TBX21 and GATA3 proteins improved. The ratio of TBX21 to GATA3 was normal, approaching the levels observed in the control group.

### 3.6 Effects of LJFE on the protein expression of Nrf2, Keap1, HO-1, and GPX4 in the spleen

**FIGURE 9:**
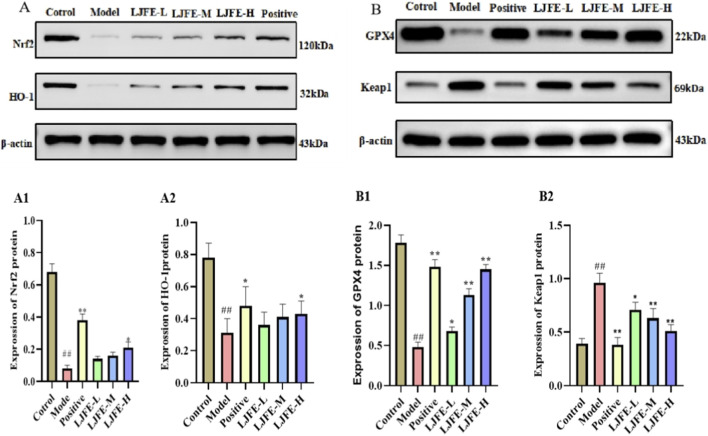
LJFE treatment activated Keap1/Nrf2/HO-1 signalling pathway-mediated oxidative stress. **(A,B)** The protein expression of Nrf2, HO-1, Keap1 and GPX4 were measured via Western blotting. **(A1)** The relative expression of Nrf2. **(A2)** The relative expression of HO-1. **(B1)** The relative expression of GPX4. **(B2)** The relative expression of Keap1. Compared with the control group, #P < 0.05, ##P < 0.01; compared with the model group, *P < 0.05, **P < 0.01.

As shown in Figure 9, compared to the control group, model mice exhibited markedly higher Keap1 protein levels in the spleen (P < 0.01), alongside significantly reduced expression of Nrf2, HO-1, and GPX4 (P < 0.05 or P < 0.01). LJFE treatment reversed these changes, decreasing Keap1 while increasing Nrf2, HO-1, and GPX4 expression—restoring them to levels comparable with the control group (P < 0.05 or P < 0.01 vs. model).

### 3.7 Molecular docking analysis of compounds from *Lonicera japonica* with Keap1/Nrf2/HO-1/GPX4 pathway proteins

**FIGURE 10:**
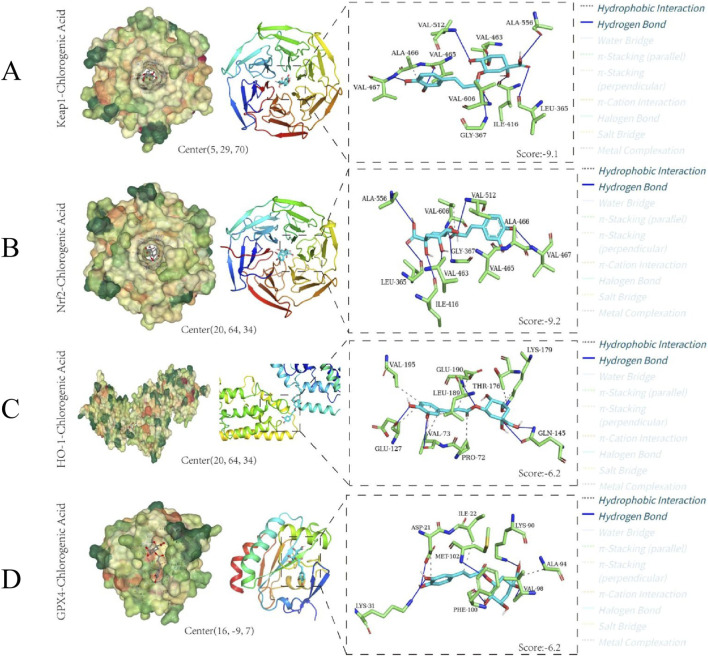
Molecular docking analysis of chlorogenic acid with Keap1, Nrf2, HO-1, and GPX4. **(A)** Binding mode and interaction pattern of chlorogenic acid with Keap1. **(B)** Binding mode and interaction pattern of chlorogenic acid with Nrf2. **(C)** Binding mode and interaction pattern of chlorogenic acid with HO-1. **(D)** Binding mode and interaction pattern of chlorogenic acid with GPX4. The left panels show the overall binding conformations of chlorogenic acid within the active pockets of the target proteins, while the right panels illustrate the key amino acid residues involved in hydrogen-bond and hydrophobic interactions. Docking scores are indicated for each protein–ligand complex.

To preliminarily explore the potential interactions between the major compounds in LJFE and oxidative stress-related target proteins, molecular docking was performed by docking 17 major compounds with Keap1, Nrf2, HO-1, and GPX4 proteins, respectively. Their binding affinities were evaluated by calculating binding energies, and the results are presented in Supplementary Table S2. The docking results indicated that the flavonoids and organic acids in LJFE exhibited good binding capabilities with all four target proteins. As a quality control marker of LJFE, chlorogenic acid showed binding energies of −9.2, −9.1, −6.2, and −6.2 kcal/mol with Nrf2, Keap1, HO-1, and GPX4, respectively, displaying stable binding conformations with all four target proteins, with the strongest affinity for Nrf2 (−9.2 kcal/mol). Figure 10 illustrates the three-dimensional docking patterns of chlorogenic acid with the four target proteins. Chlorogenic acid binds to key amino acid residues in the active sites of the target proteins through non-covalent interactions, including hydrogen bonds, hydrophobic interactions, and π-cation interactions.

### 3.8 LJFE modulates Keap1/Nrf2/HO-1 pathway and anti-inflammatory activity in RAW264.7 cells (in vitro)

**FIGURE 11:**
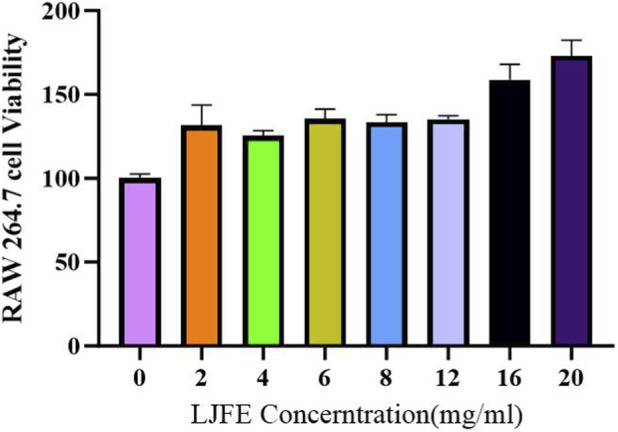
Effect of LJFE on the viability of RAW264.7 cells determined by CCK-8 assay.

The CCK-8 assay was used to evaluate the cytotoxicity of LJFE on RAW264.7 cells at concentrations of 2–20 mg/mL (Figure 11). The results showed that LJFE had no obvious cytotoxicity in the tested concentration range, and the cell viability was maintained at the level equivalent to or slightly higher than that of the control group. To avoid non-specific stimulation caused by extremely high concentrations and observe the dose-dependent effect of LJFE, 4 mg/mL, 8 mg/mL and 16 mg/mL were selected as the low, medium and high dose gradients for subsequent *in vitro* experiments, which was consistent with the experimental design of *in vivo* animal experiments.

**FIGURE 12:**
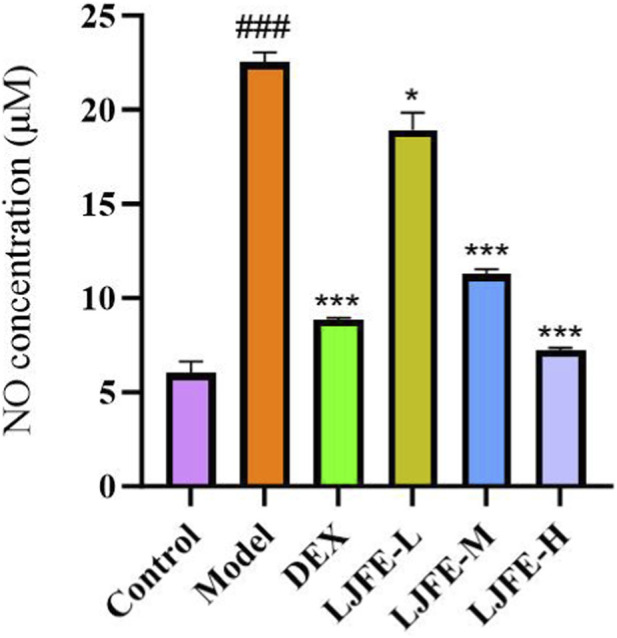
Effect of LJFE on nitric oxide (NO) secretion in LPS-induced RAW264.7 cells.

NO is a key inflammatory mediator, and its excessive release is a typical characteristic of macrophage activation under inflammatory stimulation. The Griess Reagent method was used to detect the NO secretion level of each group (Figure 12). Compared with the blank control group, LPS stimulation significantly promoted the release of NO in RAW264.7 cells (P < 0.01), indicating the successful construction of the inflammatory cell model. Compared with the LPS group, DEX as a positive control significantly inhibited the production of NO (P < 0.01). After co-treatment with different concentrations of LJFE under LPS induction, the NO secretion level of RAW264.7 cells decreased gradually with the increase of LJFE concentration, and the NO levels in the LJFE-M and LJFE-H groups were significantly lower than those in the LPS group (P < 0.05 or P < 0.01), showing a clear dose-dependent trend.

**FIGURE 13:**
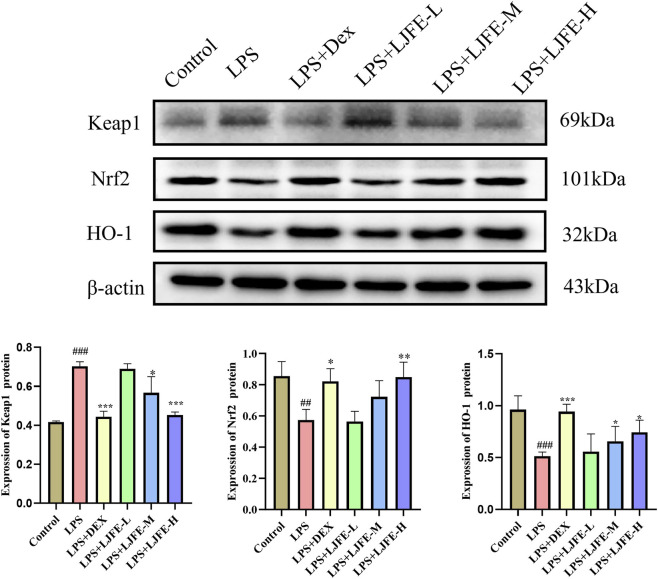
Western blot analysis was performed to evaluate the protein expression levels of Keap1, Nrf2, HO-1. Quantitative analysis demonstrated significant changes in protein expression, indicating that LJFE modulates the Keap1/Nrf2/HO-1 signaling pathway and attenuates oxidative stress. Compared with the control group, ##P < 0.01 and ###P < 0.001; compared with the model group, *P < 0.05, **P < 0.01 and ***P < 0.001.

Western blot was used to detect the expression levels of Keap1, Nrf2 and HO-1 proteins in RAW264.7 cells of each group (Figure 13). The results showed that after LPS stimulation, the expression of Keap1 protein was significantly upregulated, while the expression of Nrf2 and HO-1 was significantly downregulated compared with the blank control group (P < 0.05 or P < 0.01). DEX treatment could downregulate the expression of Keap1 and restore the protein levels of Nrf2 and HO-1 to a certain extent. After co-incubation with LJFE in the LPS-induced immune inflammation model, the expression of Keap1 protein was gradually downregulated with the increase of LJFE concentration, while the expression of Nrf2, HO-1 proteins was upregulated in a dose-dependent manner, and the differences in the LJFE-M and LJFE-H groups were more significant compared with the LPS group (P < 0.05 or P < 0.01). These *in vitro* experimental results were consistent with the *in vivo* findings, suggesting that LJFE may directly regulate the Keap1/Nrf2/HO-1 signalling pathway in immune cells and improve the oxidative stress state under inflammatory conditions. However, given that some individual compounds in LJFE are known PAINS (see Discussion), these *in vitro* data should be interpreted with caution.

## 4 Discussion

In this study, we established an immunosuppressed mice model to investigate the beneficial effects of the nonpolysaccharide fraction from the aqueous extract of LJFE on immune health. We further examined its influence on the expression of key immune-related proteins to better understand its regulatory role. Our findings suggest that LJFE may enhance immune function by modulating oxidative stress and related cellular processes. These results confirm that LJFE alleviates CTX-induced thymic/spleen atrophy and tissue damage, laying a structural foundation for immune function recovery.

Oxidative stress and immune suppression are closely interlinked in immunosuppressed models (Avila-Nava et al., 2022). Excessive free radicals, such as reactive oxygen species (ROS), can impair immune cell viability and trigger programmed cell death, particularly ferroptosis (Wang et al., 2023; Wang J. et al., 2020). Therefore, strengthening antioxidant defenses and alleviating oxidative stress represent promising strategies for restoring immune homeostasis (Wang D. et al., 2020). In this context, our findings indicate that LJFE enhances immune function partly by modulating oxidative stress and ferroptosis processes. Specifically, in the CTX-induced immunosuppression model, the increase in immunoglobulin levels likely indicates a general restoration of humoral immune function. Furthermore, antioxidant factors such as SOD and GSH help maintain the body’s healthy state and improve immune function by scavenging ROS, MDA, and other oxidative stress-generated substances, thereby regulating immune balance, alleviating inflammatory responses, and promoting tissue repair. Consistently, our biochemical data demonstrated that LJFE significantly increased SOD activity and GSH levels while reducing MDA levels. These results indicate that LJFE enhances the antioxidant capacity of immunosuppressed mice, reduces oxidative stress responses, and alleviates CTX-induced cellular damage.

Initially, we utilized UPLC-MS/MS analysis to characterize the major chemical compounds of LJFE, including phenolic acids and flavonoids, which are consistent with the quality control markers of LJF specified in the Chinese Pharmacopoeia. These findings indicate that LJFE contains these compounds as its main chemical constituents (Liu et al., 2024).

To validate our hypothesis, we focused on the impact of LJFE on the relevant pathways involved in oxidative stress in immunosuppressed mice (Guan et al., 2022). Western blot analysis showed that LJFE downregulated the expression of Keap1 and upregulated the expression of Nrf2, HO-1, and the ferroptosis inhibitor GPX4 in spleen tissue (Han Y. et al., 2022; Li et al., 2023; Pratelli et al., 2022). Upon assessing the levels of antioxidant factors in the serum, we found that LJFE significantly increased the activity of SOD, the levels of GSH and reduced MDA levels (Dai et al., 2020; Zhang C. et al., 2022). These protein expression changes, together with the biochemical improvements, support the conclusion that LJFE ameliorates immunosuppression by modulating the Keap1/Nrf2/HO-1/GPX4 pathway.

Notably, through metabolomics analysis, among the differential metabolites, we found that the differential metabolism of hydroferulic acid showed significant differences compared with that of the normal group and the model group. It is speculated that hydroferulic acid may be produced by the metabolism of chlorogenic acid in LJFE (Cruz-Carrión et al., 2022). Pathway enrichment analysis based on the KEGG database further indicated that chlorogenic acid and its metabolites are associated with four pathways related to antioxidative stress and immune enhancement. These findings highlight the importance of dietary phytochemicals from *L. japonica* Flos in modulating host metabolism, reducing oxidative stress, and ultimately supporting immune health.

In the Glutamatergic Synapse metabolic pathway, ferulic acid, a secondary metabolite of chlorogenic acid, has antioxidant and neuroprotective effects (El Gizawy and Boshra, 2023; Ioana et al., 2022). It can alleviate nerve damage by inhibiting the excitotoxicity of glutamate (Xiaolong et al., 2022). The compound L-tryptophan is metabolized into 5-HT, which indirectly regulates the balance of glutamatergic signalling (Xiaolong et al., 2022). The Glutamatergic Synapse pathway is involved in the synthesis of GSH (Sara et al., 2022), inhibits the generation of ROS (Tokio et al., 2022), relieves nerve cell damage (Liran et al., 2022), and enhances immune function. In the Vitamin B6 metabolism pathway, the Vitamin B6 metabolite PLP is involved in regulating the *in vivo* metabolism of the compounds L-tryptophan, L-tyrosine, and L-phenylalanine (Cellini et al., 2020; Sheeba et al., 2024). L-tyrosine and L-phenylalanine, as precursors of dopamine (Hang et al., 2022), affect synaptic function through the dopamine-glutamate interaction and are involved in the Glutamatergic Synapse metabolic pathway (Lia et al., 2023). Secondly, the Vitamin B6 metabolic pathway is involved in the synthesis of GSH (Yuan et al., 2024), scavenges free radicals in the body, and also enhances immunity by regulating hormone levels (Qing et al., 2025).

In the Drug Metabolism—Other Enzymes pathway, secondary metabolites of chlorogenic acid—such as caffeic acid, 4-coumaric acid, and ferulic acid—are metabolized by COMT/SULT enzymes, which prolong their antioxidant activity (Liu et al., 2024). Meanwhile, the drug metabolism—other enzymes pathway is also involved in the synthesis of GSH and scavenges ROS (Lucie et al., 2013; Tokio et al., 2022). Ferulic acid, caffeic acid, and 4-coumaric acid all have strong antioxidant activities (Haleh et al., 2022), which reduce the consumption of GSH and GPX4 and decrease the ferroptosis mediated by oxidative stress. In the metabolic pathway of the ferroptosis pathway, ferulic acid, caffeic acid, and 4-coumaric acid are also involved in the metabolism of cysteine, a substrate for GSH synthesis (Ge et al., 2024; Krax et al., 2025; Laura et al., 2022), indirectly affecting the synthesis of GSH. In addition, the literature reports that these acids can also activate the Nrf2 signalling pathway (Haoming et al., 2023; Li et al., 2024; Sabitha et al., 2020), upregulate the expression of GCL, GS, and GPX4 (Li et al., 2022; Yun et al., 2023), improve the ability to resist oxidative stress, inhibit the ferroptosis mediated by oxidative stress, and enhance immune function.

It should be noted that molecular docking is a computational prediction and does not provide experimental evidence of direct binding or *in vivo* target engagement. Nevertheless, molecular docking techniques were employed to explore the interactions between chlorogenic acid and target proteins, including Keap1, Nrf2, HO-1, and GPX4. The results revealed that chlorogenic acid can bind stably to these target proteins through various noncovalent interactions, particularly through the strongest binding affinity to Nrf2. Based on these computational predictions, it is speculated that chlorogenic acid could enhance cellular antioxidant defense capabilities by stabilizing the Nrf2 structure or activating its downstream signalling pathway (Jiashe et al., 2024). Additionally, the predicted strong binding between chlorogenic acid and Keap1 may inhibit Keap1-mediated Nrf2 degradation, thereby indirectly improving Nrf2 activity and regulating the antioxidant pathway (Renaud et al., 2022). However, given that chlorogenic acid is a PAINS compound, these predictions require experimental validation.

It is important to note that chlorogenic acid, luteolin, and quercetin are classified as pan-assay interference compounds (PAINS). Such compounds can produce artifactual results in certain *in vitro* assays and computational docking due to non-specific interactions (e.g., aggregation, redox activity). Therefore, the *in vitro* data (Section 3.8) and molecular docking predictions (Section 3.7) presented in this study should be interpreted with caution. The primary conclusions of this work are based on *in vivo* experiments using the whole extract LJFE, which are not invalidated by the PAINS properties of individual constituents. Future studies using orthogonal assays (e.g., target-based screens with counterscreens) are required to validate the specific contributions of each compound.

Previous studies have reported that the major compounds of LJFE—chlorogenic acid, luteolin, and quercetin—may activate the Nrf2/HO-1 pathway and exert antioxidant and immunomodulatory effects. For example, chlorogenic acid enhances antioxidant enzymes via Nrf2-HO-1 signalling and suppresses inflammation (Rashidi et al., 2022). Luteolin has been reported to activate Keap1/Nrf2 by covalent modification of Keap1 cysteines, upregulating HO-1 and reducing oxidative stress (Hayakawa et al., 2025; Liang et al., 2025). Quercetin has been suggested to inhibits ferroptosis through the Nrf2/HO-1/GPX4 axis (Feng et al., 2023). Nevertheless, all three compounds are classified as PAINS; therefore, the literature data, especially from *in vitro* assays, should be interpreted with caution, and the conclusions from the present study are preliminary.

In the *in vitro* experiments, LJFE treatment significantly inhibited excessive NO release in LPS-stimulated RAW264.7 cells, suggesting its potential role in regulating inflammatory responses. Furthermore, LJFE dose-dependently downregulated Keap1 and upregulated Nrf2 and HO-1 expression in these cells, which was highly consistent with the *in vivo* results. These *in vitro* findings are consistent with the possibility that LJFE may directly affect the Keap1/Nrf2/HO-1 signalling pathway in immune cells and improve the oxidative stress state under inflammatory conditions. However, due to the PAINS properties of some individual components, these *in vitro* results are not conclusive and require orthogonal validation.

Several limitations should be acknowledged. First, the CTX-induced immunosuppression mouse model, while well-established, does not fully recapitulate the heterogeneity and complexity of human immunosuppression. Therefore, the therapeutic effects observed in these models may not directly translate to clinical settings in humans. Second, this study focused on short-term (10-day) efficacy; the long-term safety and efficacy of LJFE remain unknown and require further investigation. Future studies using chronic models and, eventually, clinical trials are necessary to establish the translational potential of LJFE. Third, while we assessed total Nrf2 protein levels and downstream targets, direct measurement of nuclear Nrf2 translocation was not performed; this should be addressed in future studies.

In summary, this study demonstrates that LJFE ameliorates CTX-induced immunosuppression by modulating the Keap1/Nrf2/HO-1/GPX4 pathway, thereby attenuating oxidative stress and ferroptosis. Our results indicate that LJFE enhances the antioxidant capacity of immunosuppressed mice, reduces oxidative stress responses, and alleviates CTX-induced cellular damage. While chlorogenic acid is a major constituent, LJFE contains multiple bioactive compounds (e.g., luteolin, quercetin) that also activate the Nrf2 pathway; therefore, the observed immunomodulatory effect likely arises from synergistic actions of these components. These findings not only elucidate the mechanistic basis of LJFE’s immunomodulatory activity but also support its potential application as a candidate for further development as a functional food ingredient for enhancing immune resilience.

## 5 Conclusion

In summary, oral administration of the nonpolysaccharide fraction of the aqueous extract of *Lonicera japonica* Flos significantly enhanced immune function in immunosuppressed mice. This effect is likely mediated through regulation of the Keap1/Nrf2/HO-1 pathway, upregulation of GPX4, and protection against oxidative stress and ferroptosis, leading to the repair of immune cells and reinforcement of immune defense. These findings suggest that *L. japonica* Flos has potential as a natural candidate for further evaluation in the context of immune support, though additional studies are needed to assess its long-term safety, efficacy in humans, and the specific roles of individual bioactive components.

## Data Availability

The mass spectrometry data generated in this study have been deposited in the MassIVE repository (https://massive.ucsd.edu) with the dataset identifier MSV000102367, and are publicly available via DOI: https://doi.org/10.25345/C59S1M06Z.
