# Supplementary material for: Nonpolysaccharide fraction of Lonicerae japonicae Flos attenuates cyclophosphamide-induced immunosuppression associated with modulation of the Keap1/Nrf2/HO-1/GPX4 signalling pathway
Source: Front Pharmacol. 2026 Jul 17;17:1853492. doi: 10.3389/fphar.2026.1853492 (PMC13424074; doi:10.3389/fphar.2026.1853492)
Supplement: Supplementary file 4 [file Table3.docx]

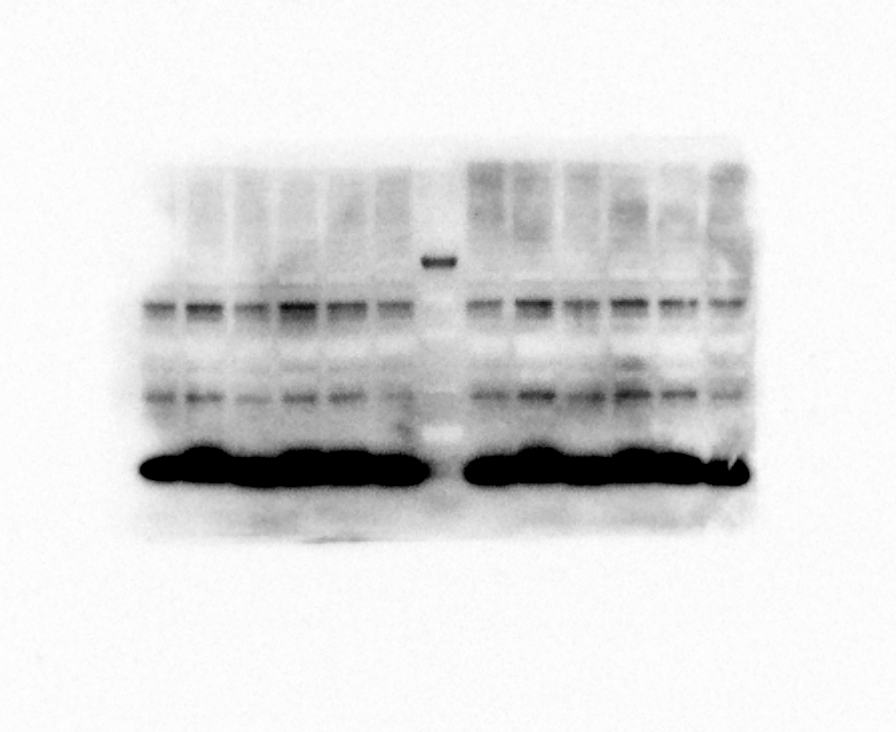

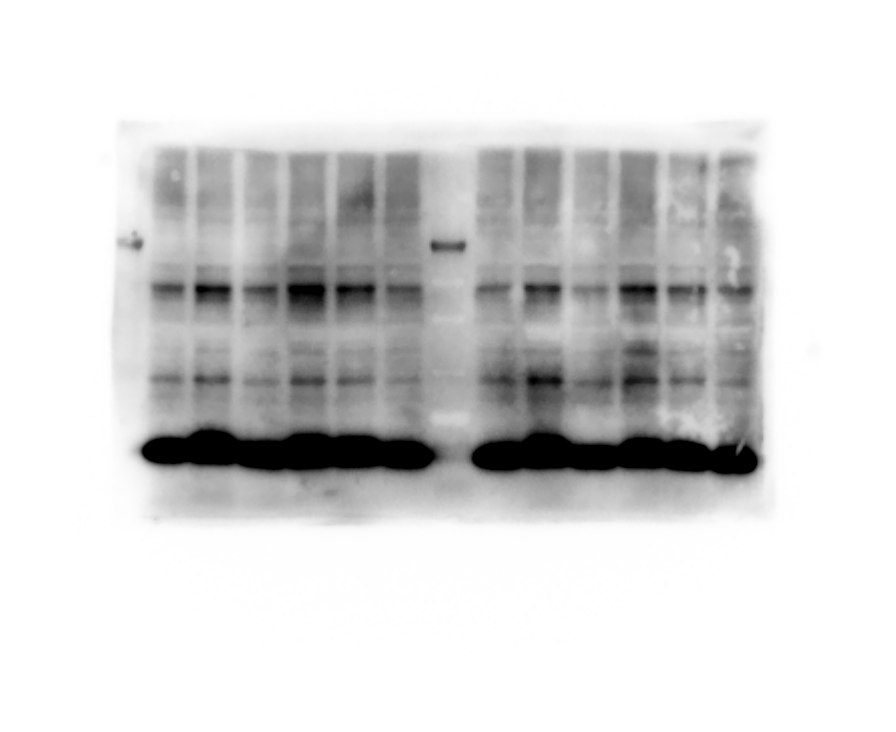
Keap1 in RAW264.7


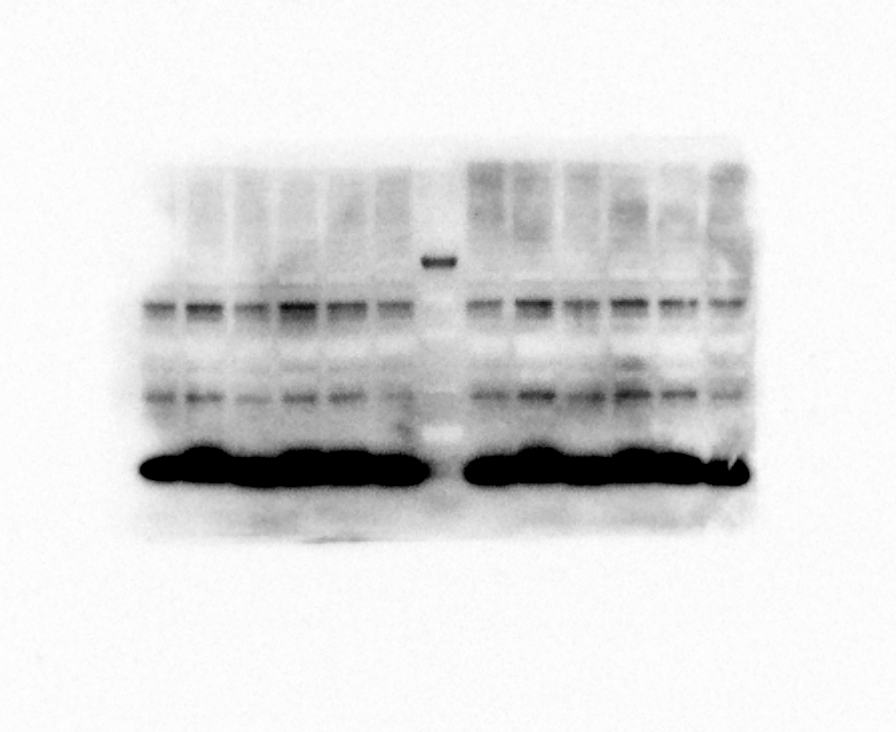


Keap1-1 Keap1-2 Keap1-3

| Control | LPS | LPS+DEX | LPS+LJFE-L | LPS+LJFE-M | LPS+LJFE-H |
| --- | --- | --- | --- | --- | --- |
| 0.42 | 0.69 | 0.46 | 0.67 | 0.50 | 0.44 |
| 0.42 | 0.69 | 0.46 | 0.68 | 0.66 | 0.45 |
| 0.41 | 0.73 | 0.41 | 0.72 | 0.54 | 0.47 |

Nrf2 in RAW264.7


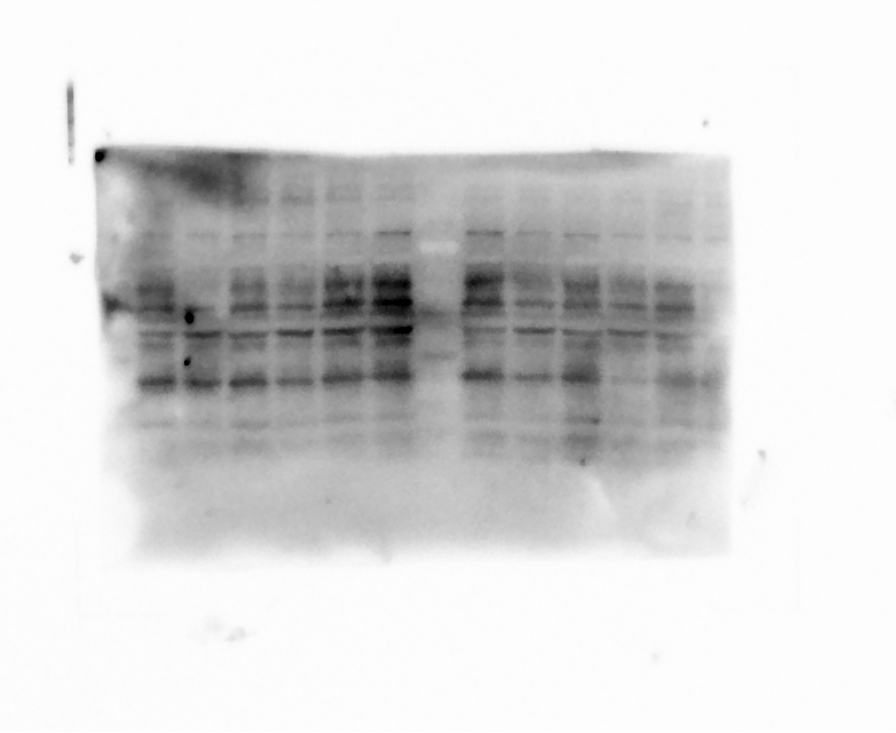

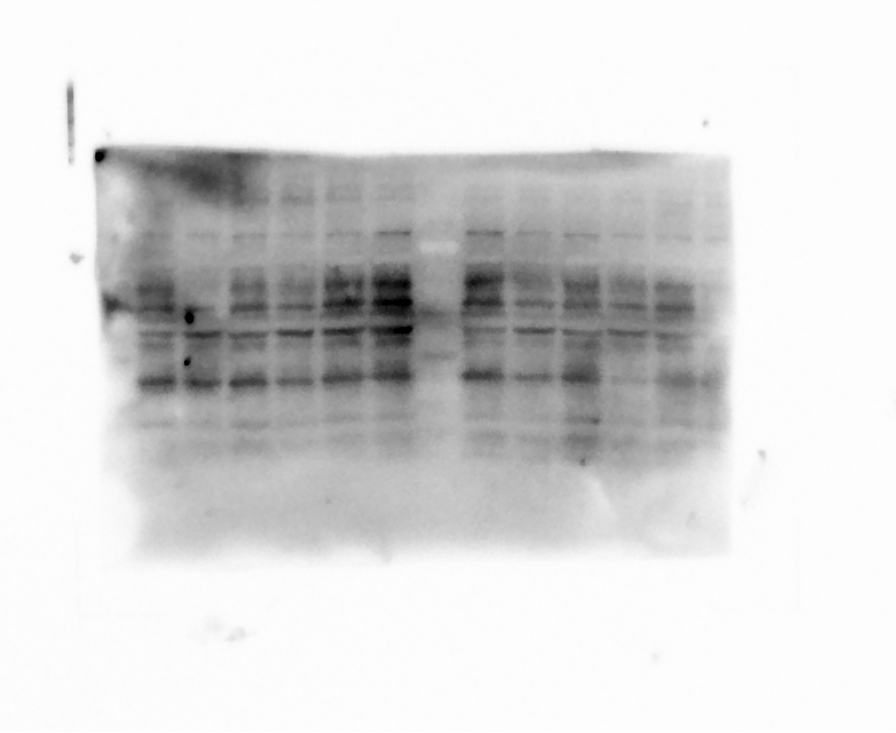

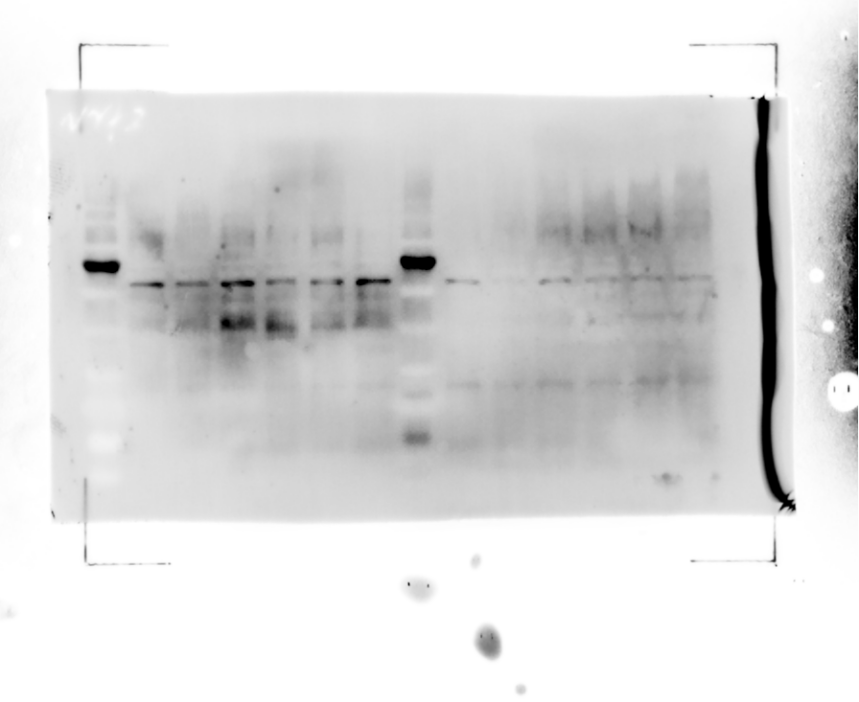


Nrf2-1 Nrf2-2 Nrf2-3

| Control | LPS | LPS+DEX | LPS+LJFE-L | LPS+LJFE-M | LPS+LJFE-H |
| --- | --- | --- | --- | --- | --- |
| 0.74 | 0.45 | 0.85 | 0.5 | 0.72 | 0.81 |
| 0.71 | 0.57 | 0.79 | 0.63 | 0.79 | 0.83 |
| 0.85 | 0.37 | 0.7 | 0.42 | 0.82 | 0.78 |

HO-1 in RAW264.7


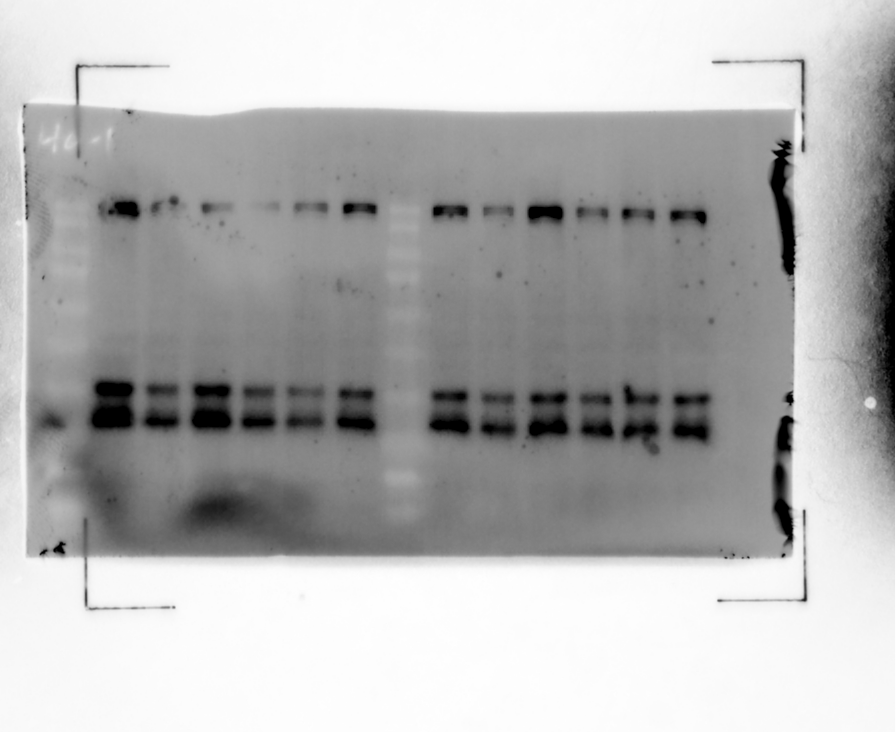


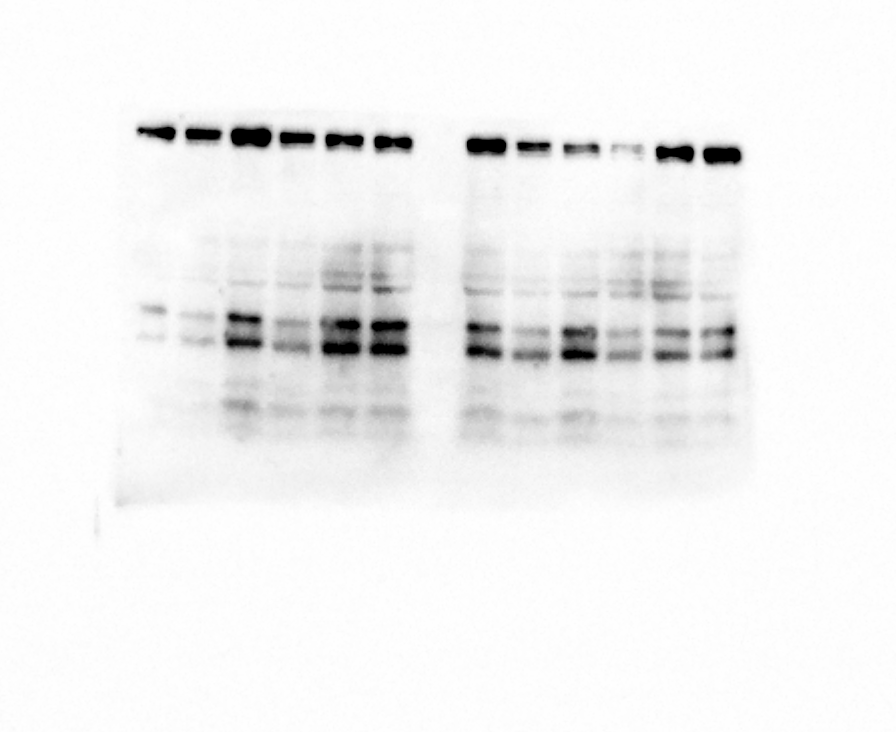

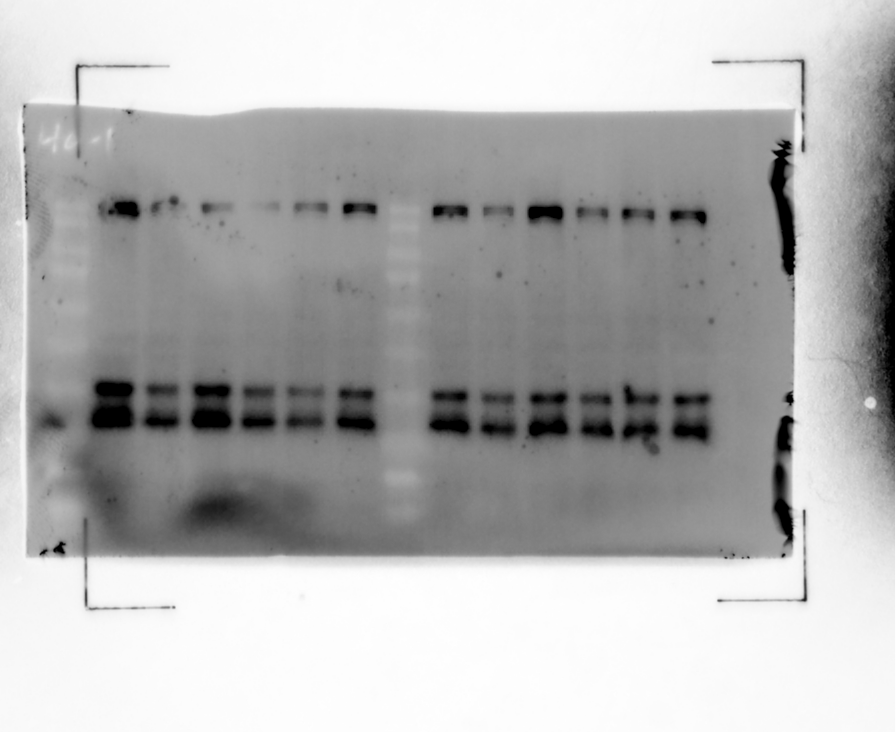


HO-1 -1 HO-1 -2 HO-1 -3

| Control | LPS | LPS+DEX | LPS+LJFE-L | LPS+LJFE-M | LPS+LJFE-H |
| --- | --- | --- | --- | --- | --- |
| 0.91 | 0.5 | 0.98 | 0.61 | 0.55 | 0.58 |
| 0.99 | 0.42 | 0.88 | 0.40 | 0.55 | 0.52 |
| 0.82 | 0.47 | 0.83 | 0.53 | 0.6 | 0.64 |


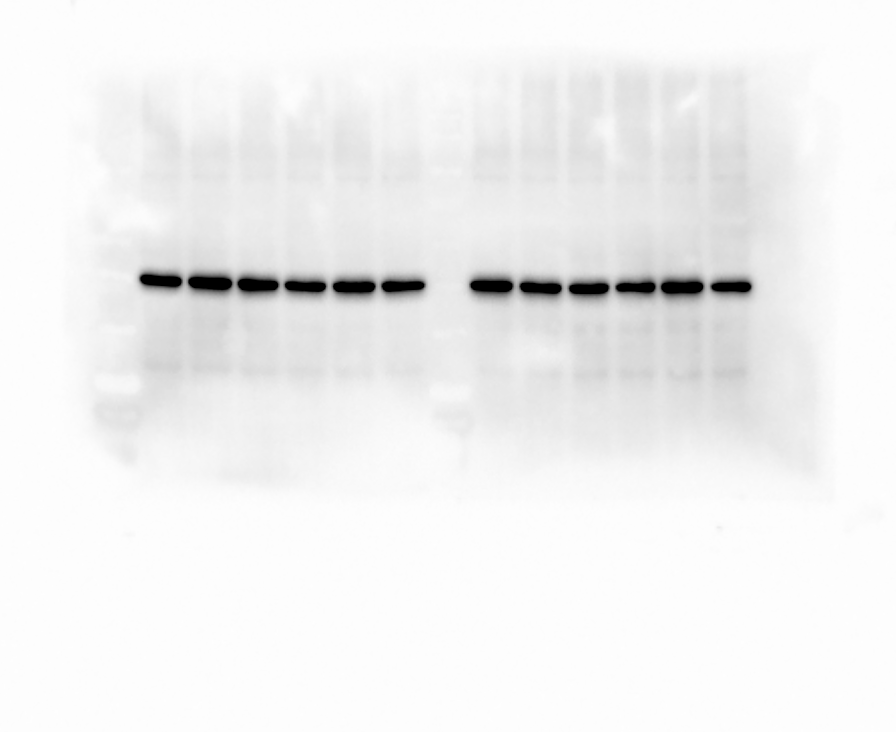
β-actin


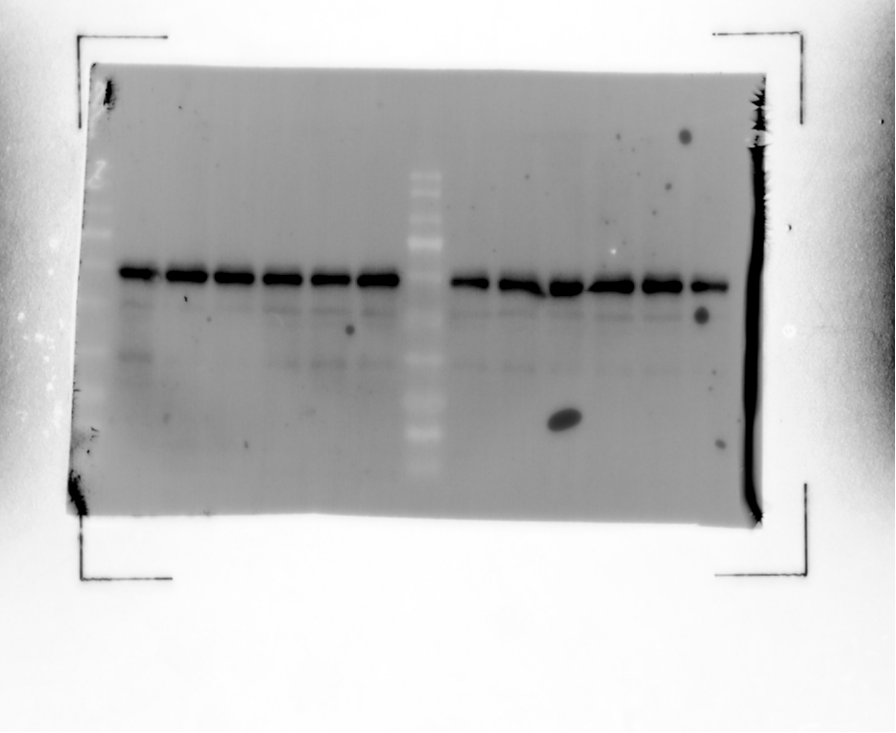

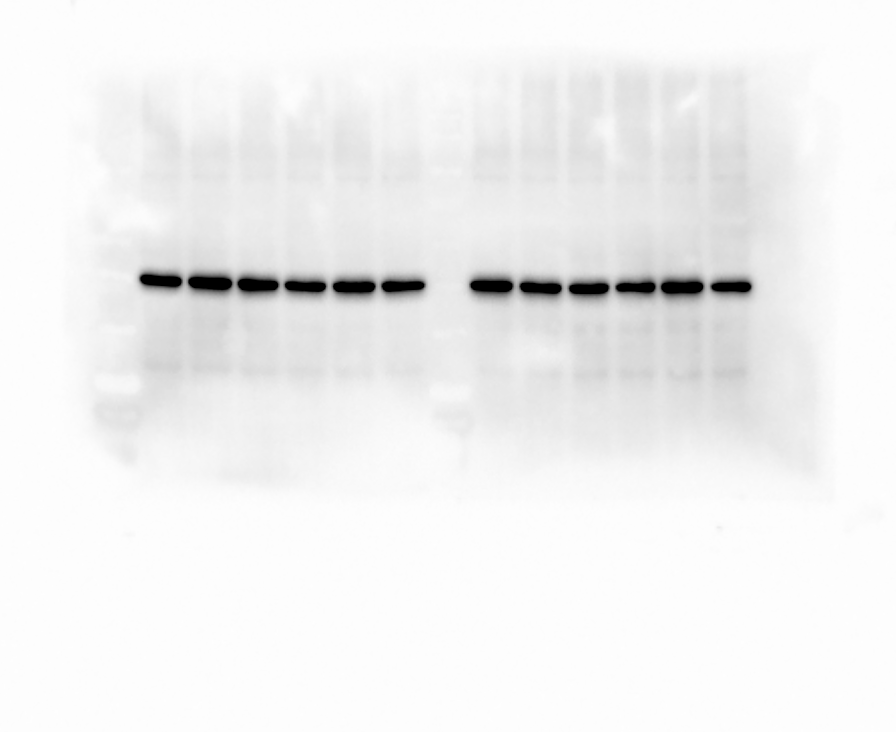


β-actin-1 β-actin-2 β-actin-3
