# Supplementary figures and images for: Nonpolysaccharide fraction of Lonicerae japonicae Flos attenuates cyclophosphamide-induced immunosuppression associated with modulation of the Keap1/Nrf2/HO-1/GPX4 signalling pathway
Source: Front Pharmacol. 2026 Jul 17;17:1853492. doi: 10.3389/fphar.2026.1853492 (PMC13424074; doi:10.3389/fphar.2026.1853492)

1. H0-1


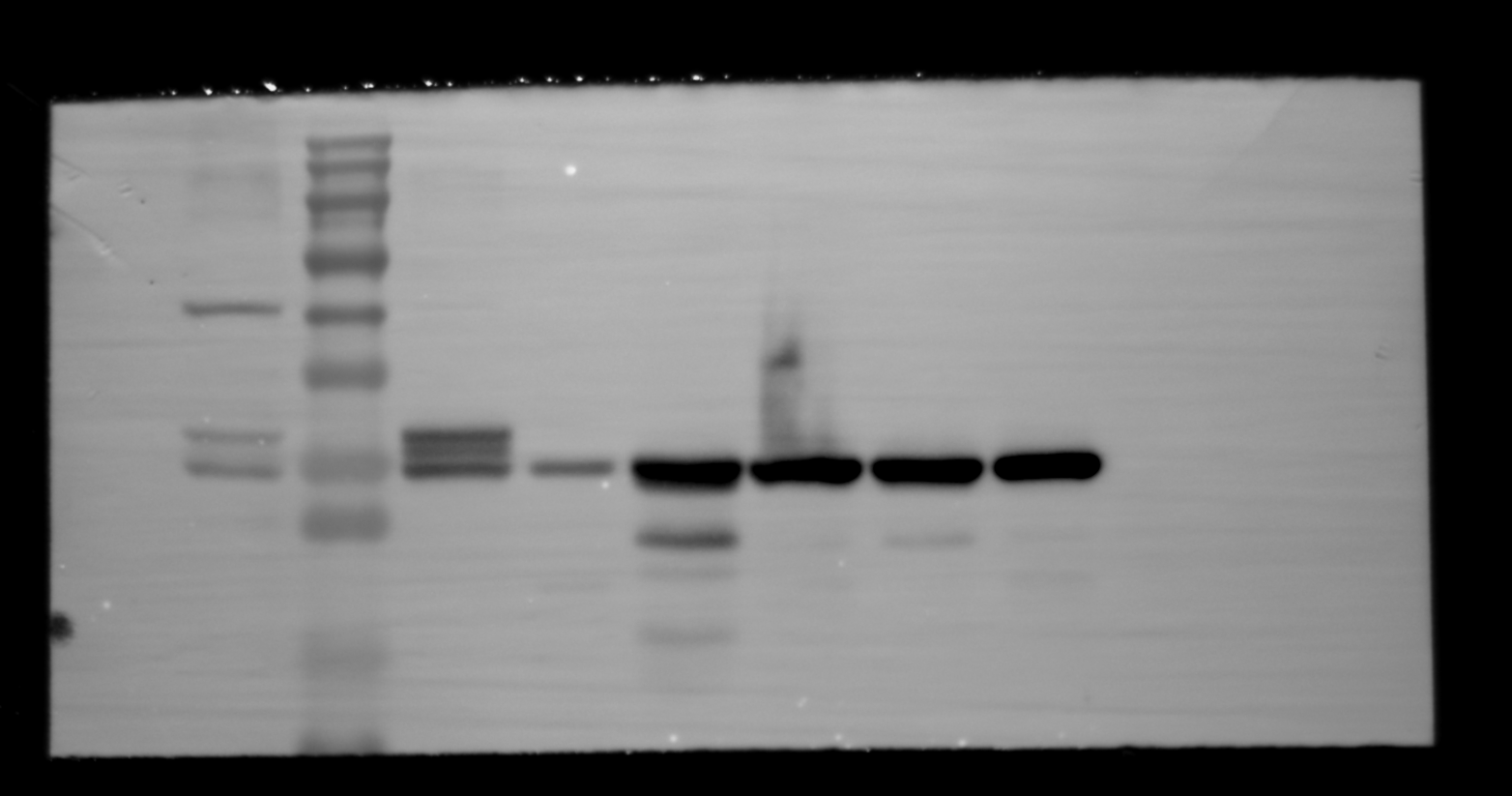

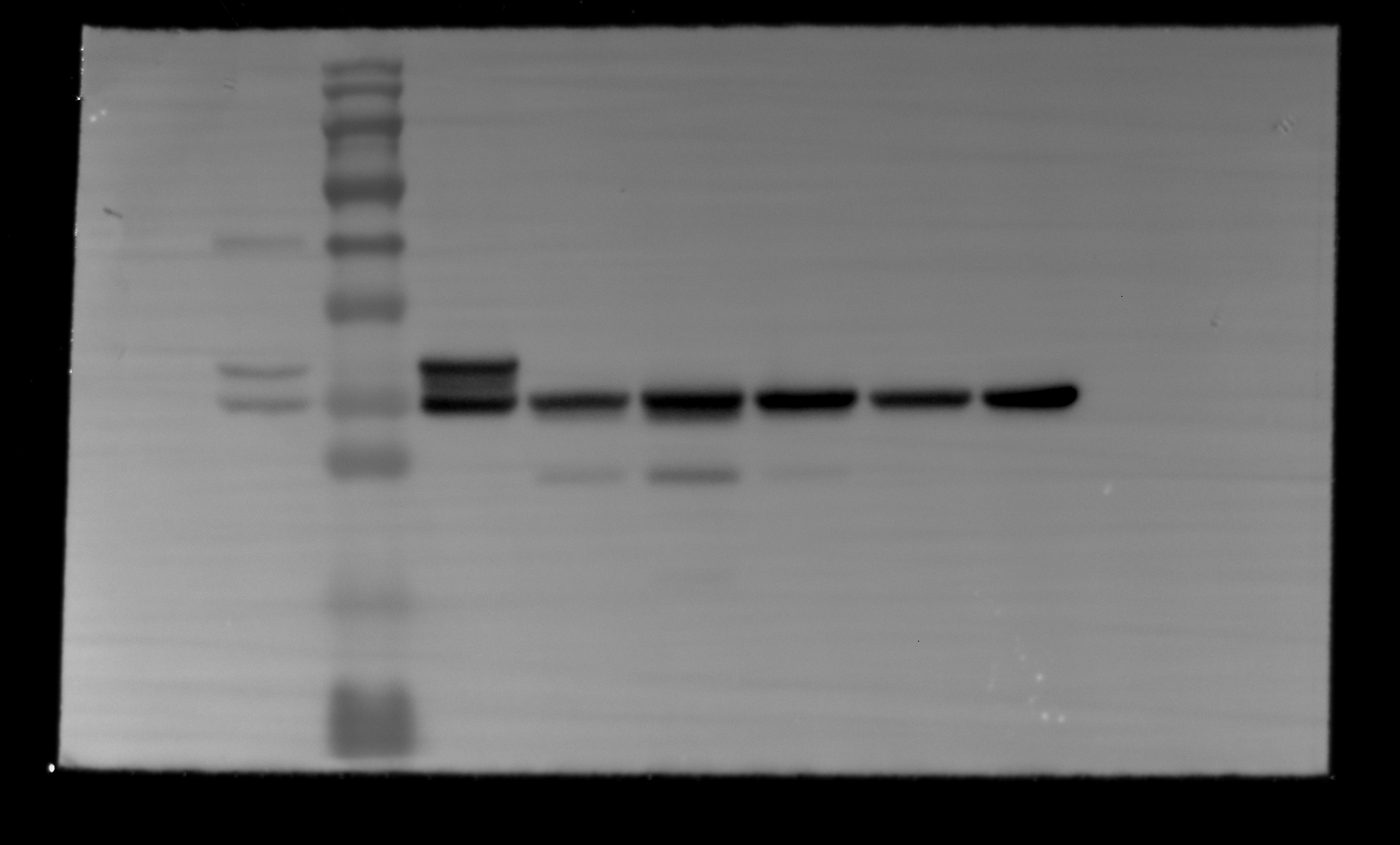


HO-1(1) HO-1(2)


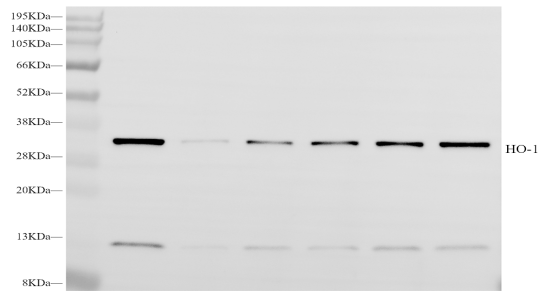

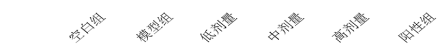


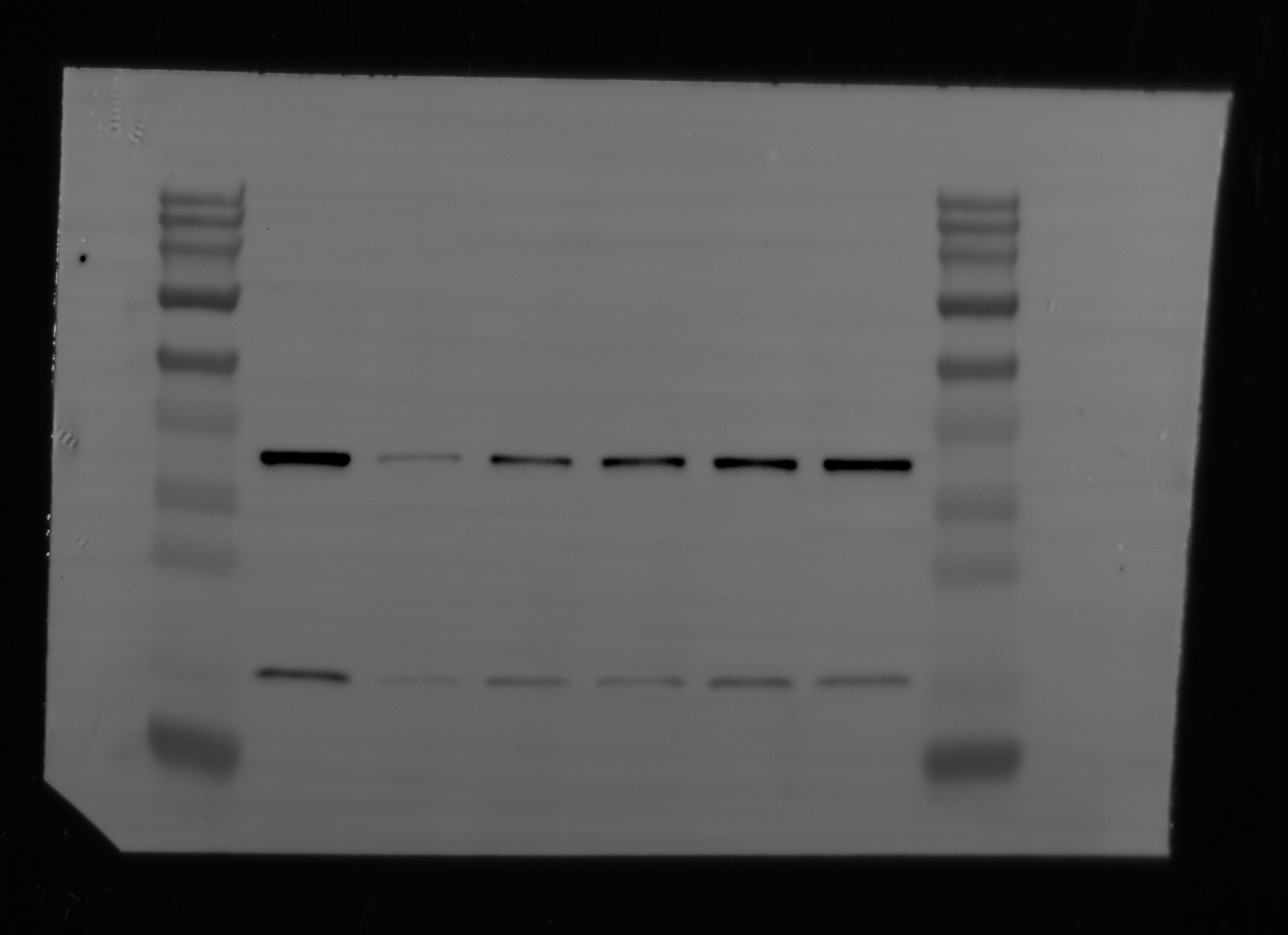


HO-1(3)

1. Nrf2


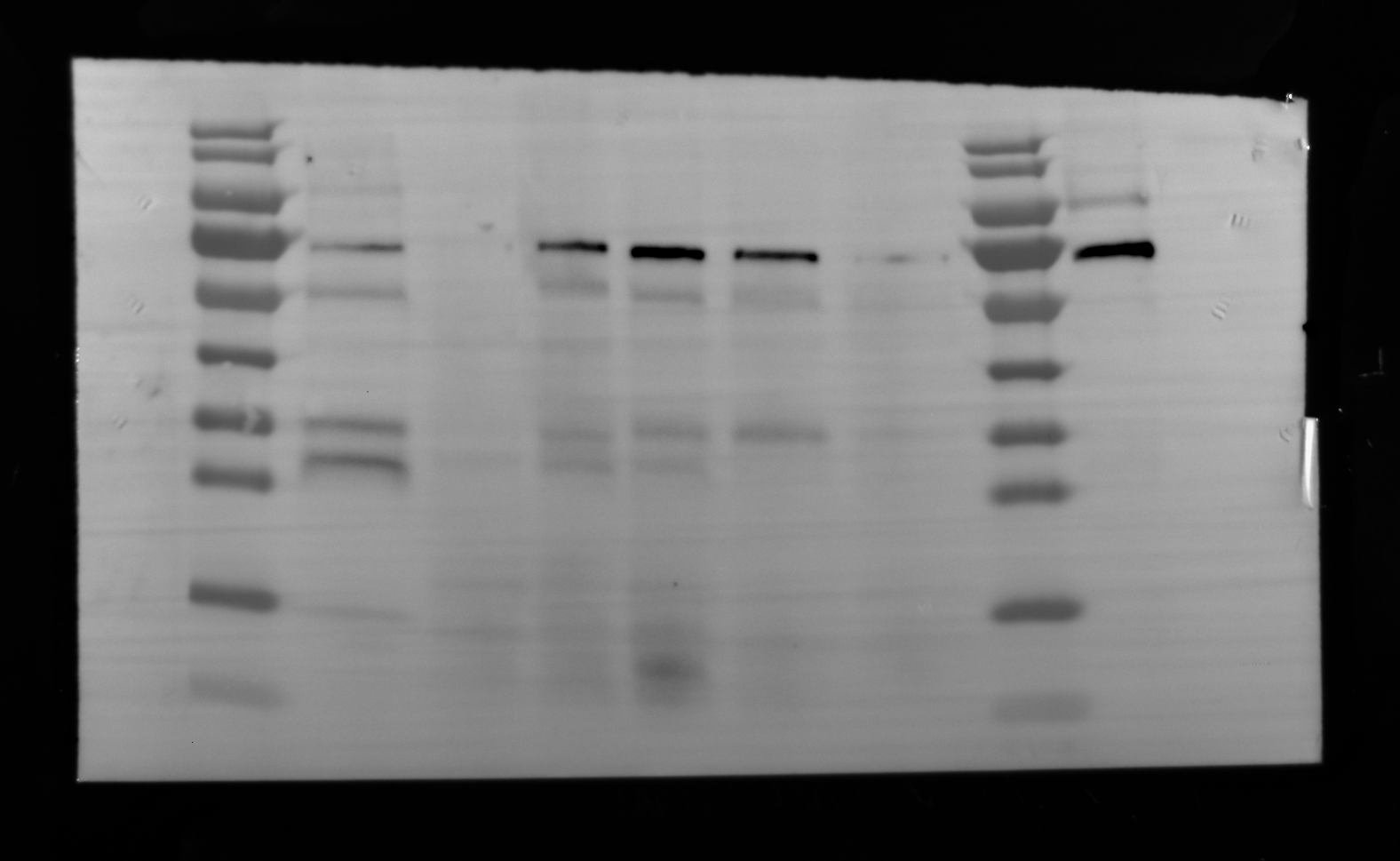

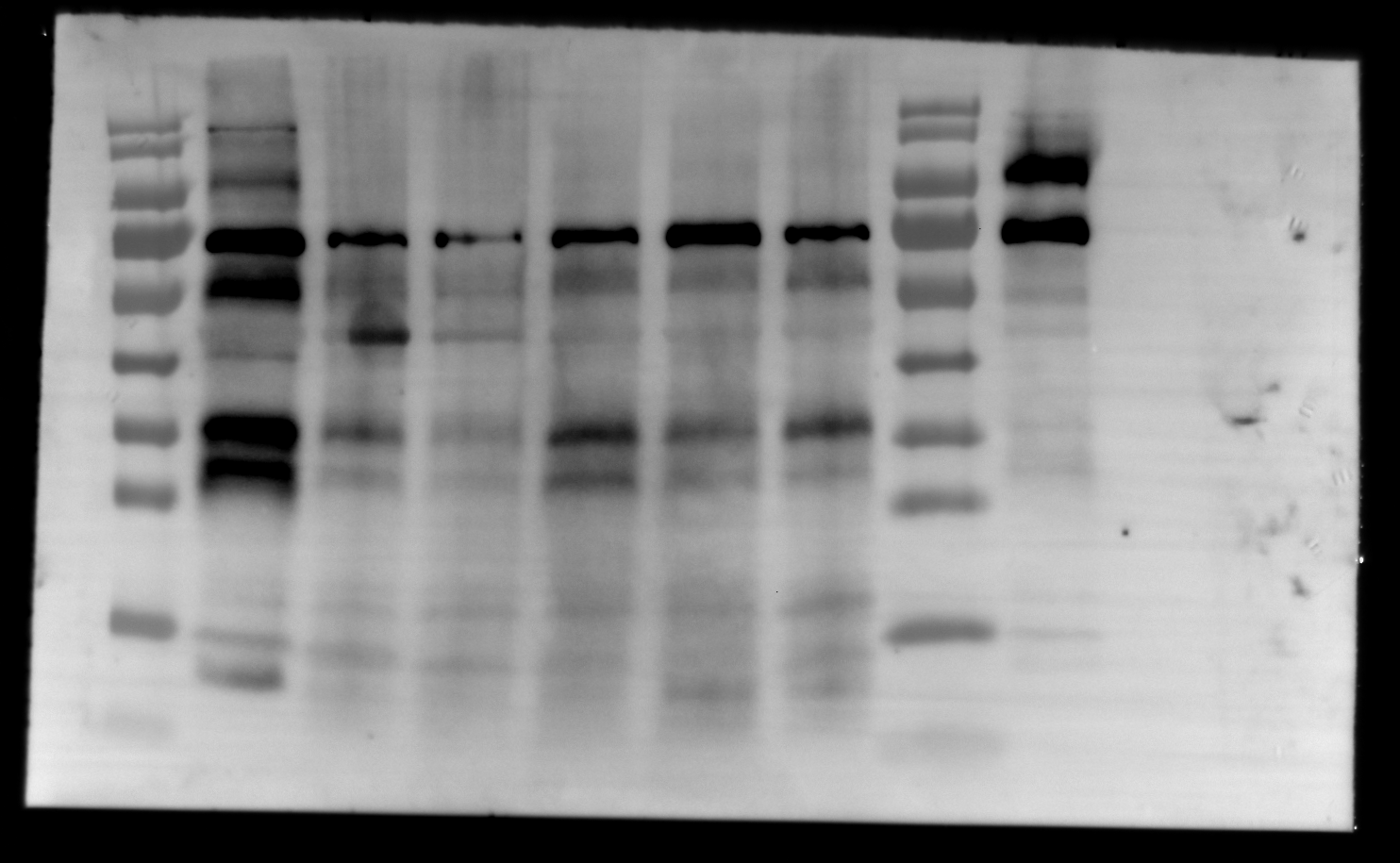


Nrf2（1） Nrf2（2）


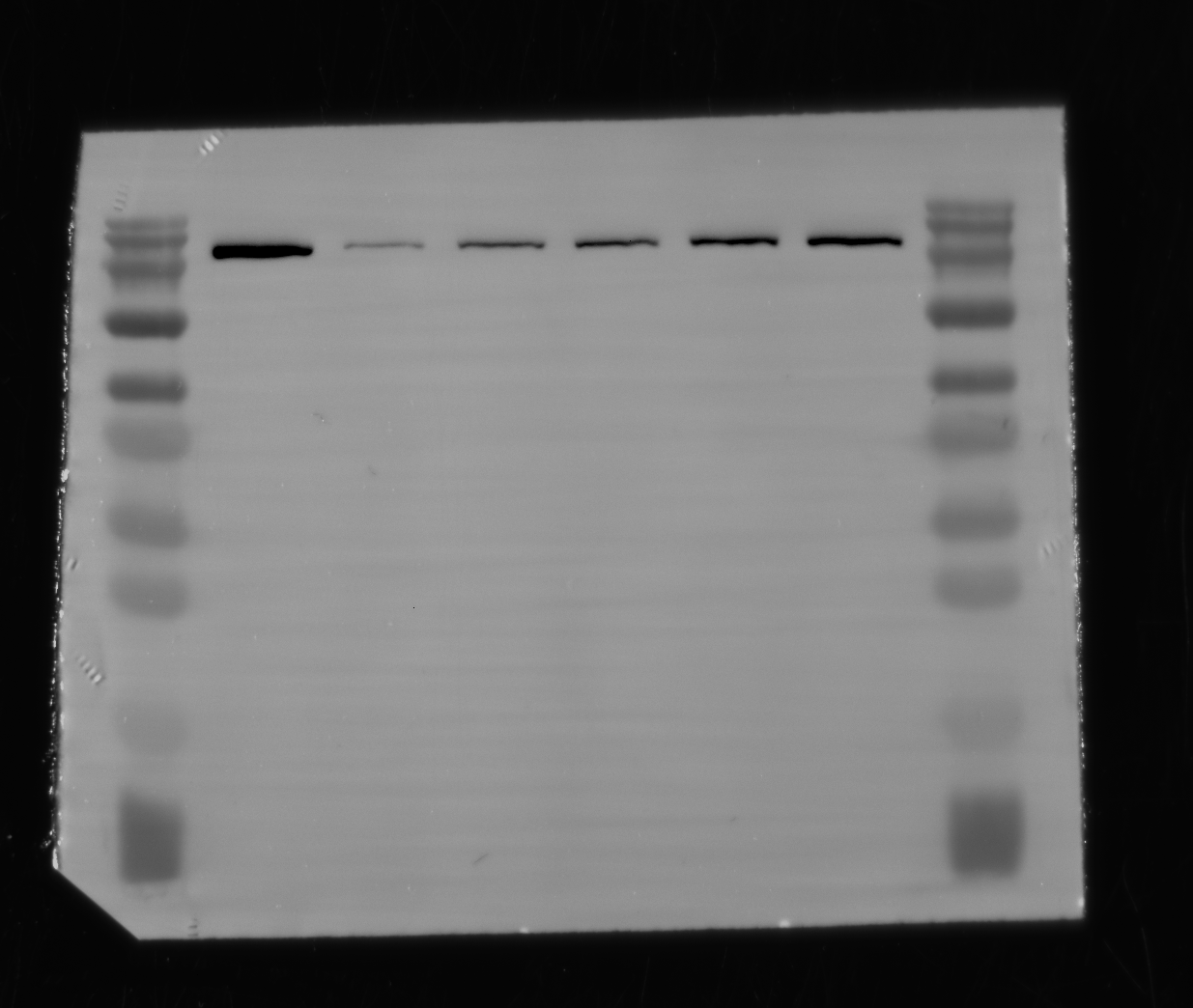

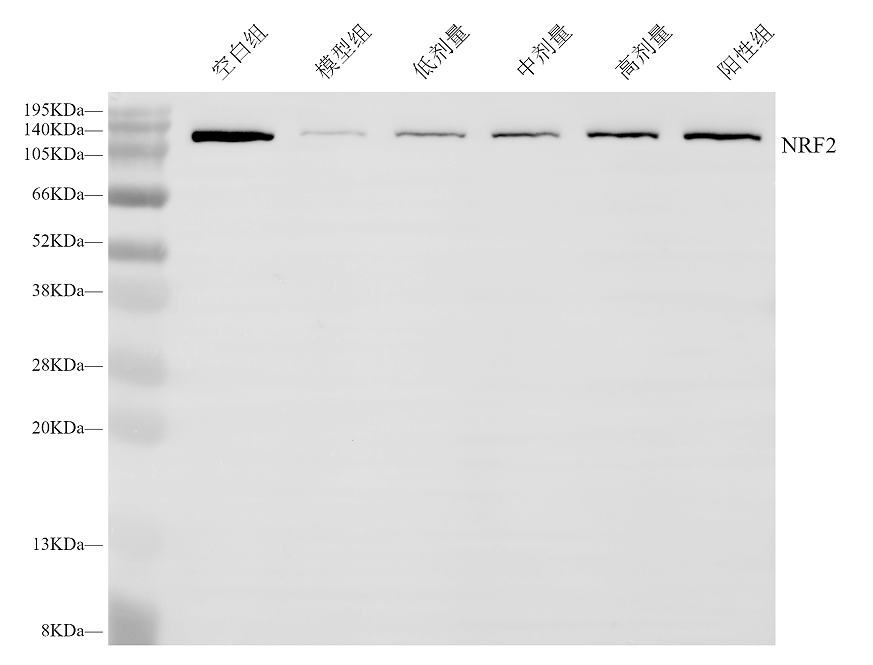


Nrf2（3）

1. β-actin


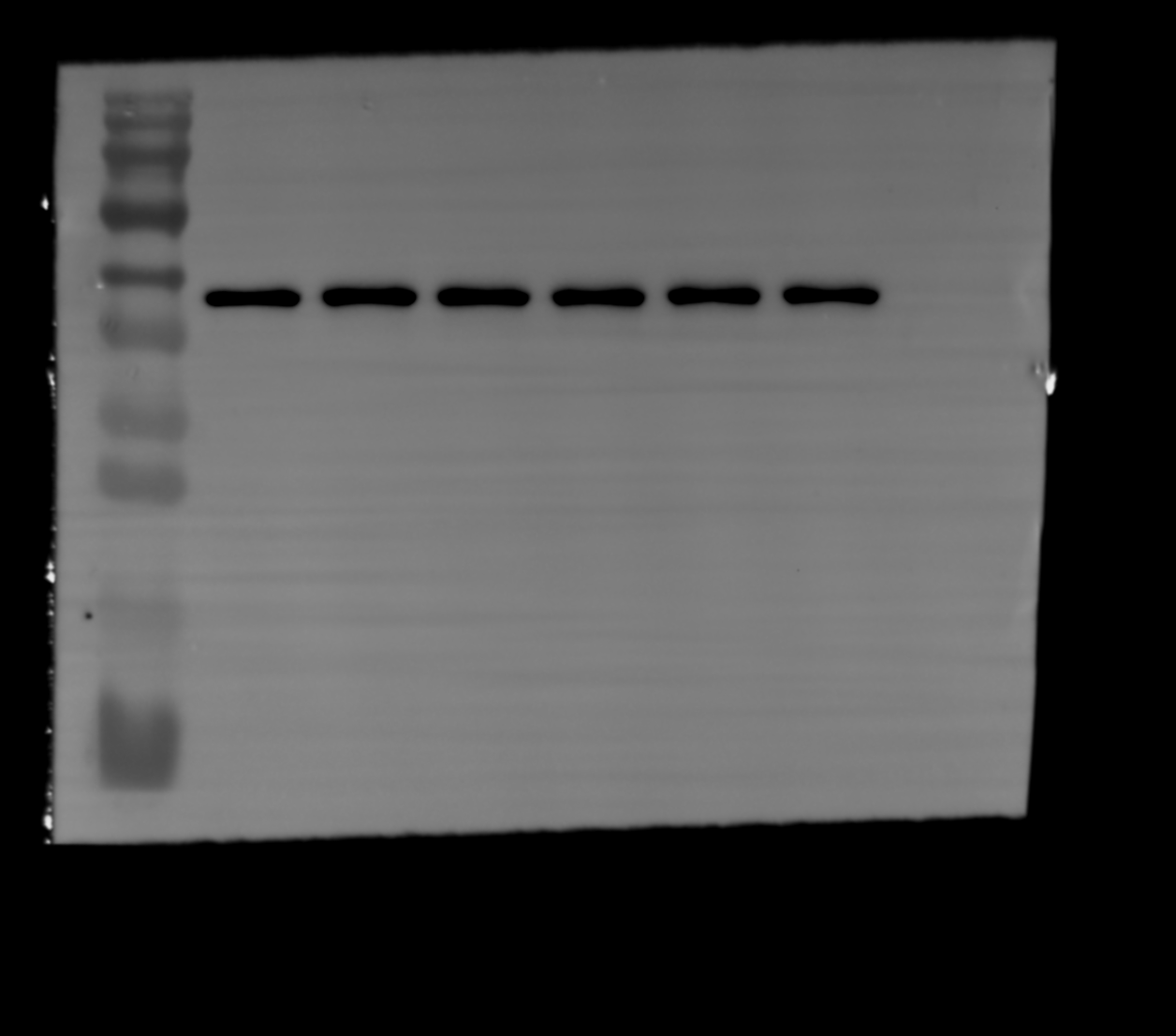

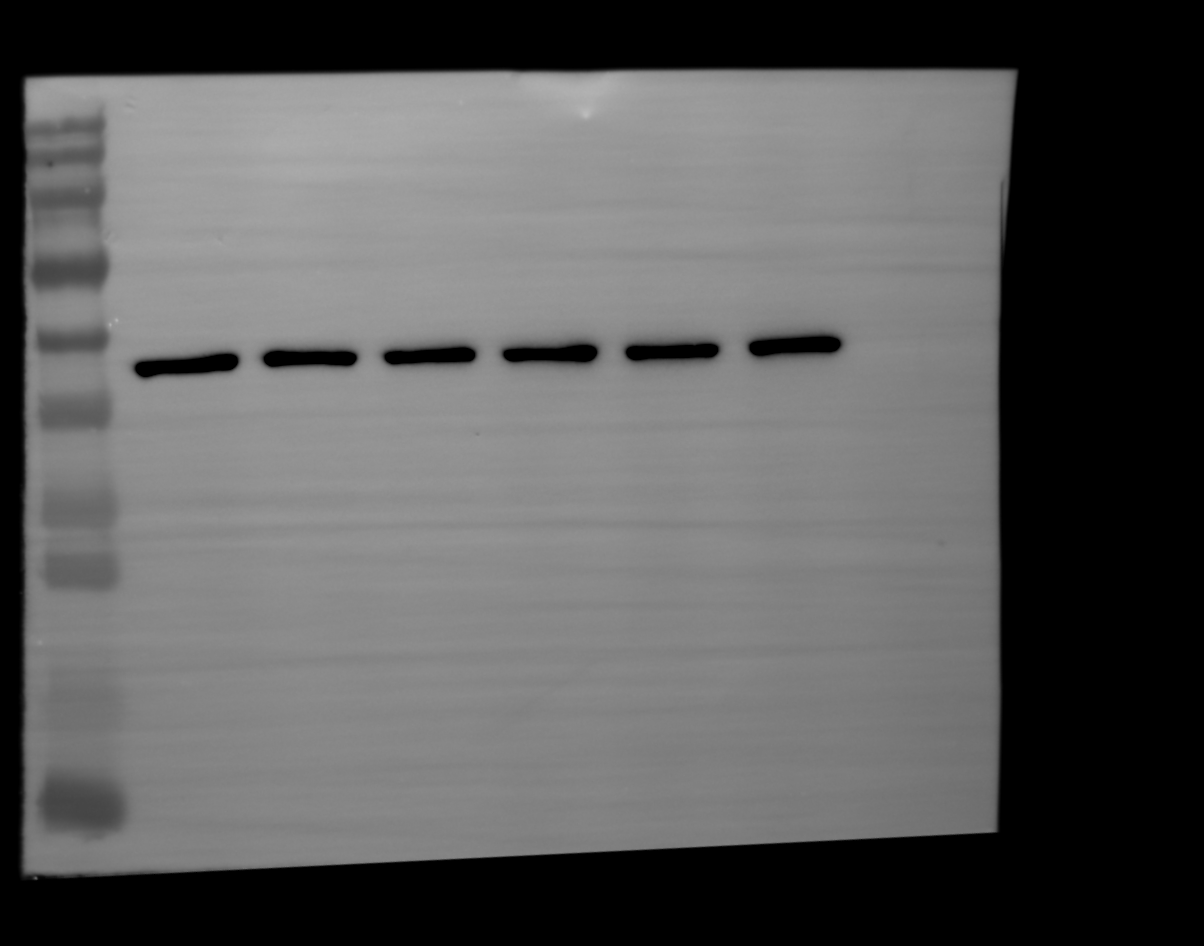

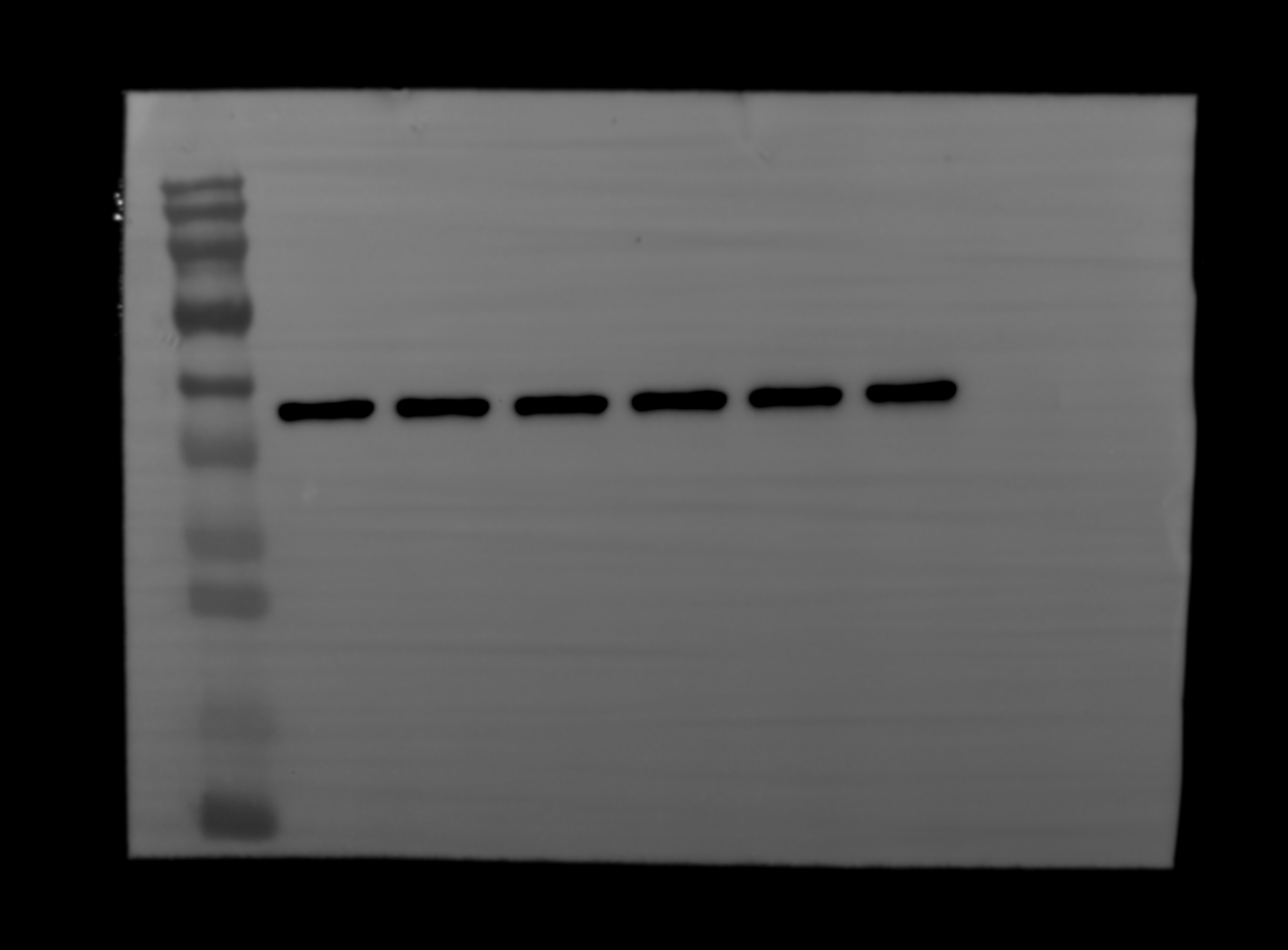

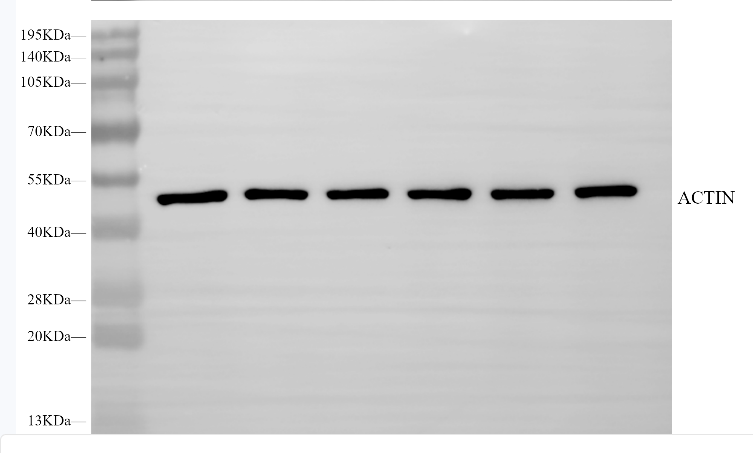


4.GPX4


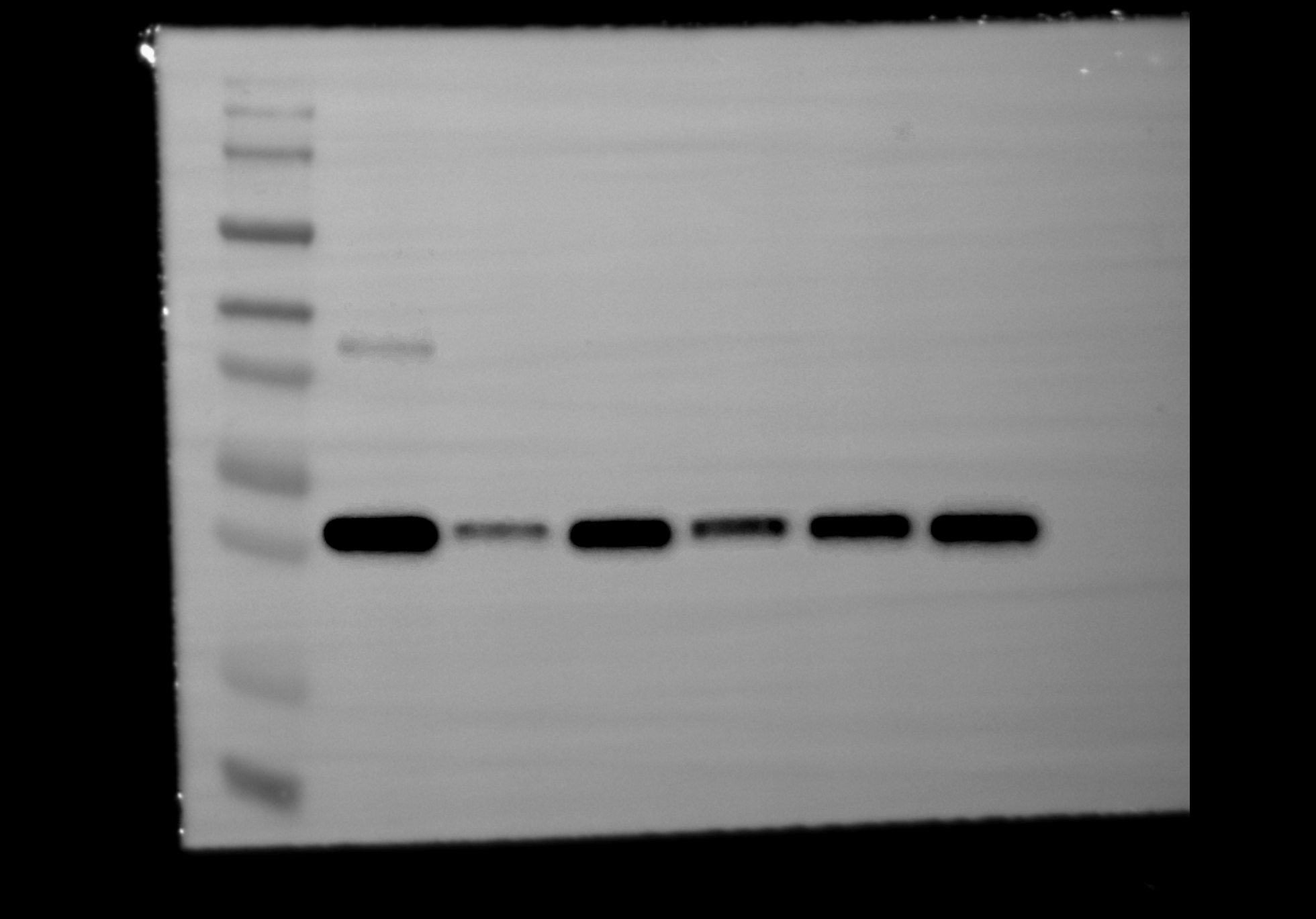

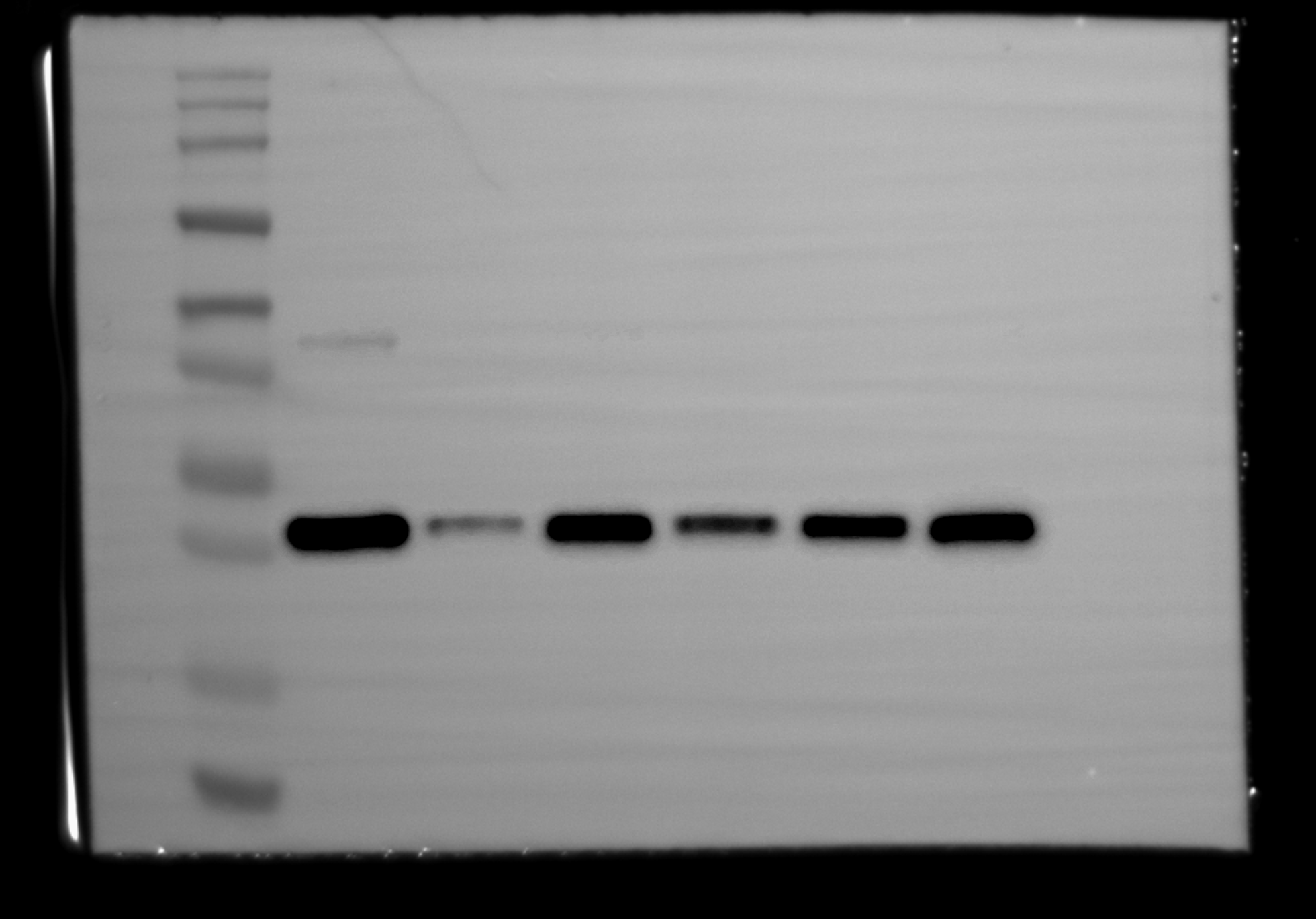


GPX4（1） GPX4（2）


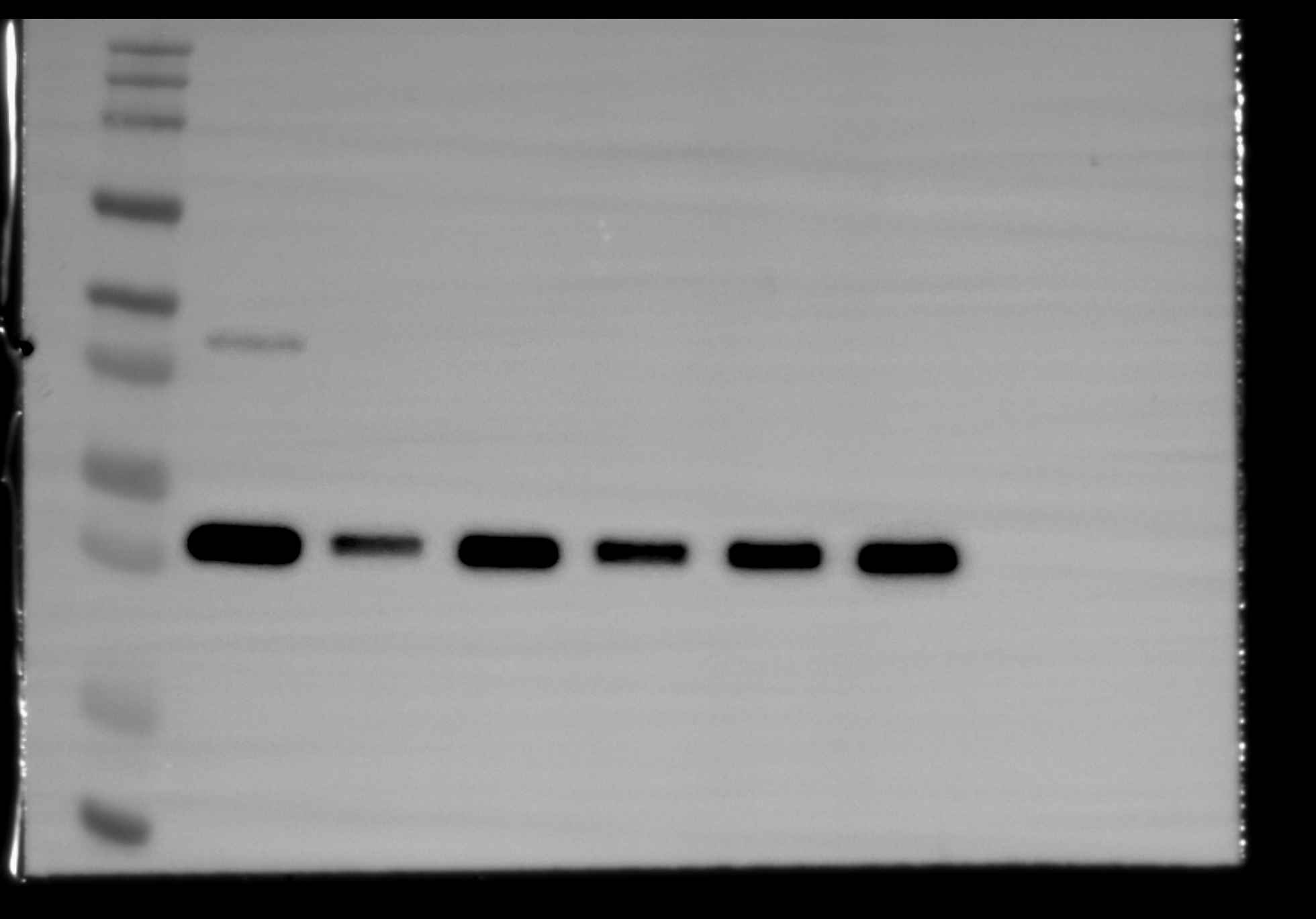

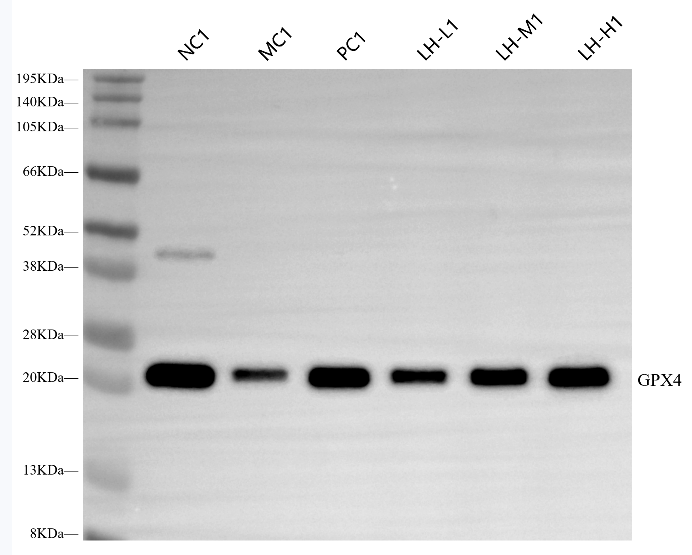


GPX4（3）

5 Keap1


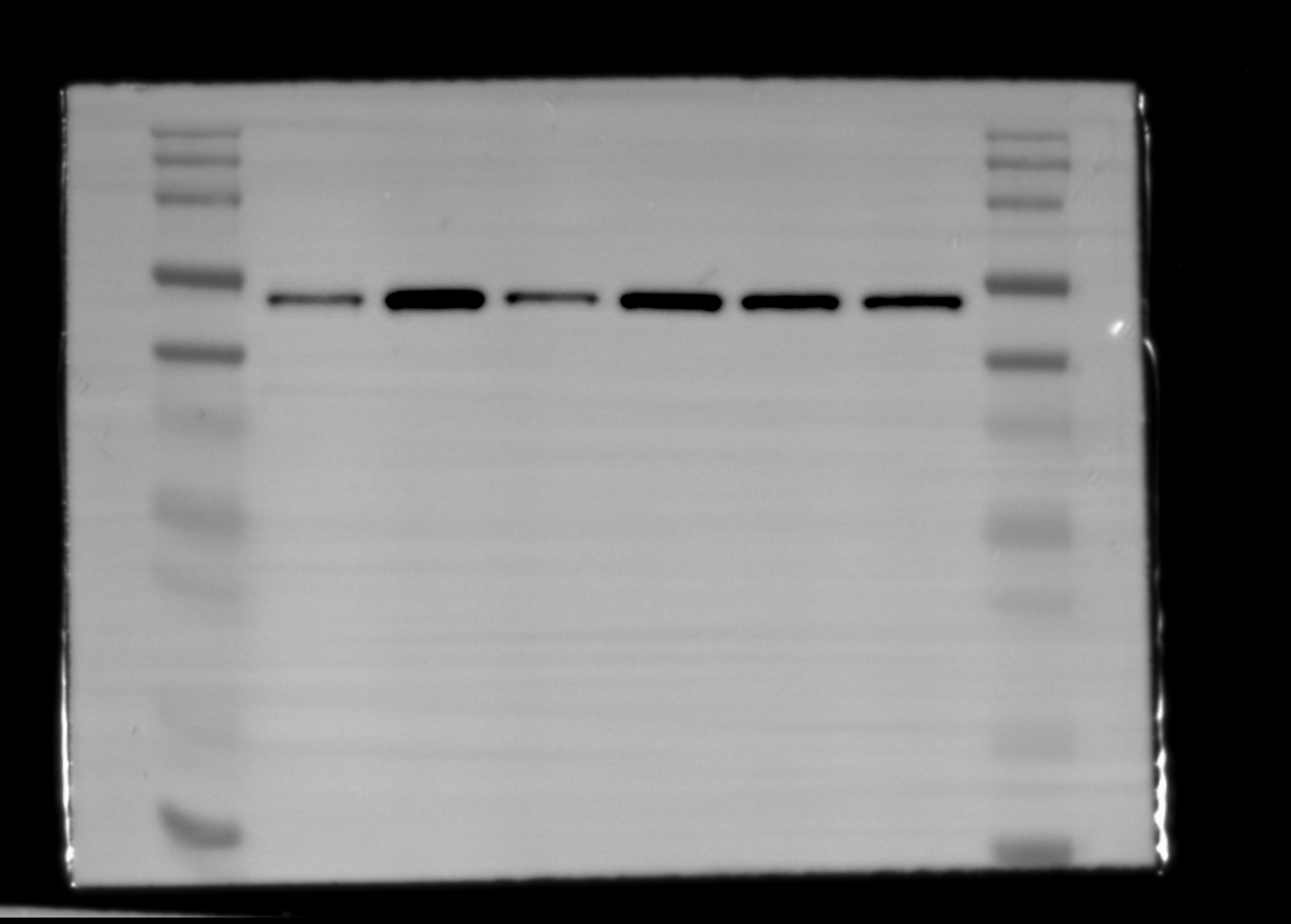

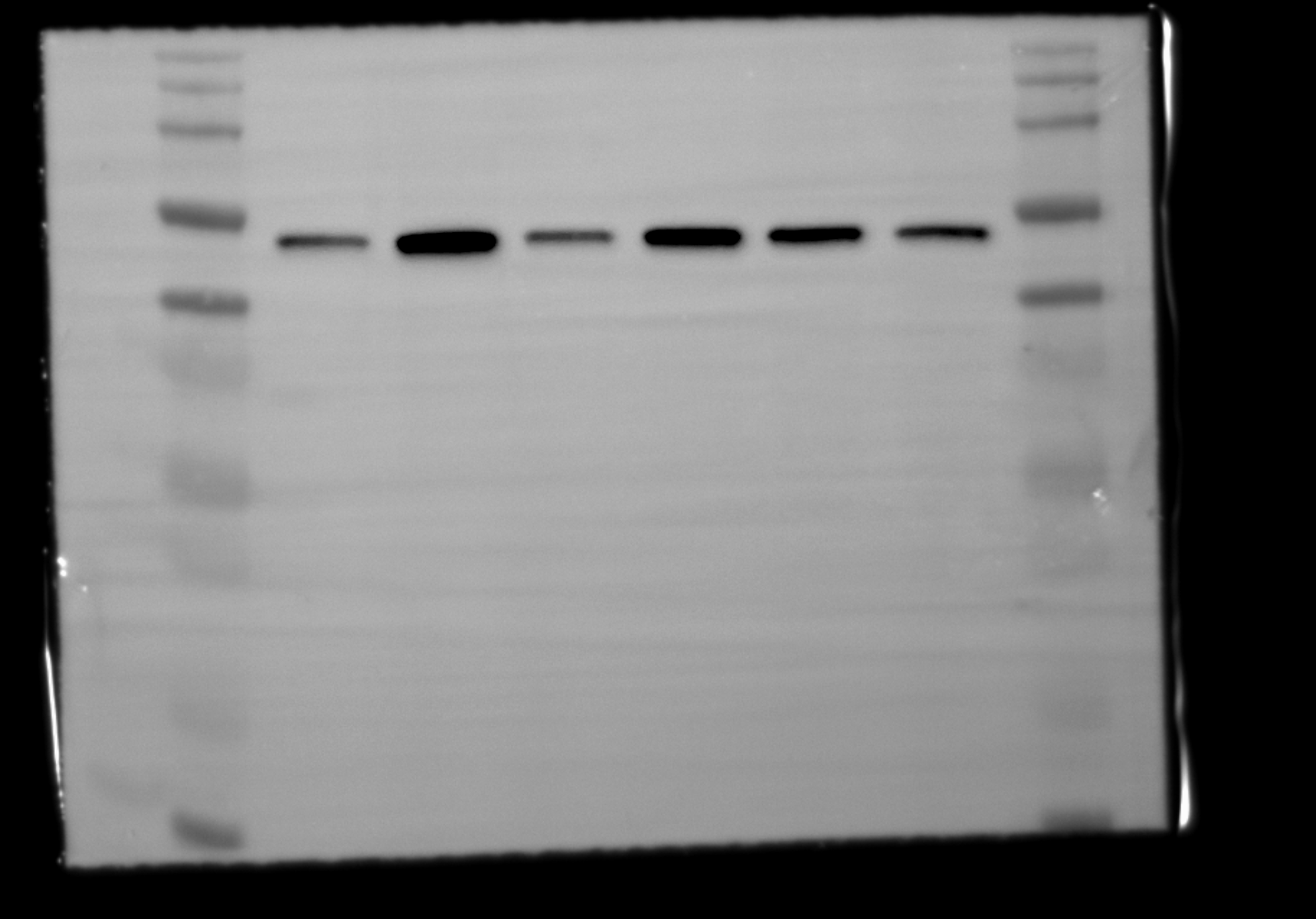


Keap1（1） keap1(2)


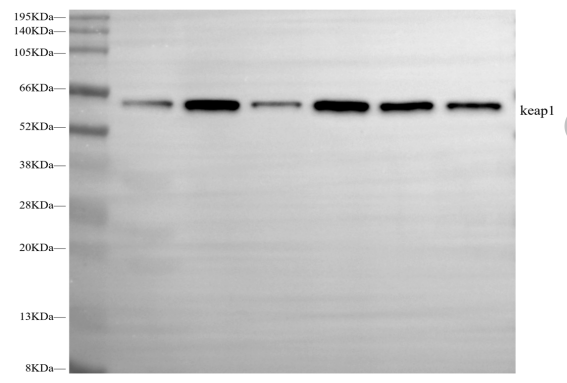

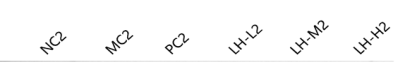


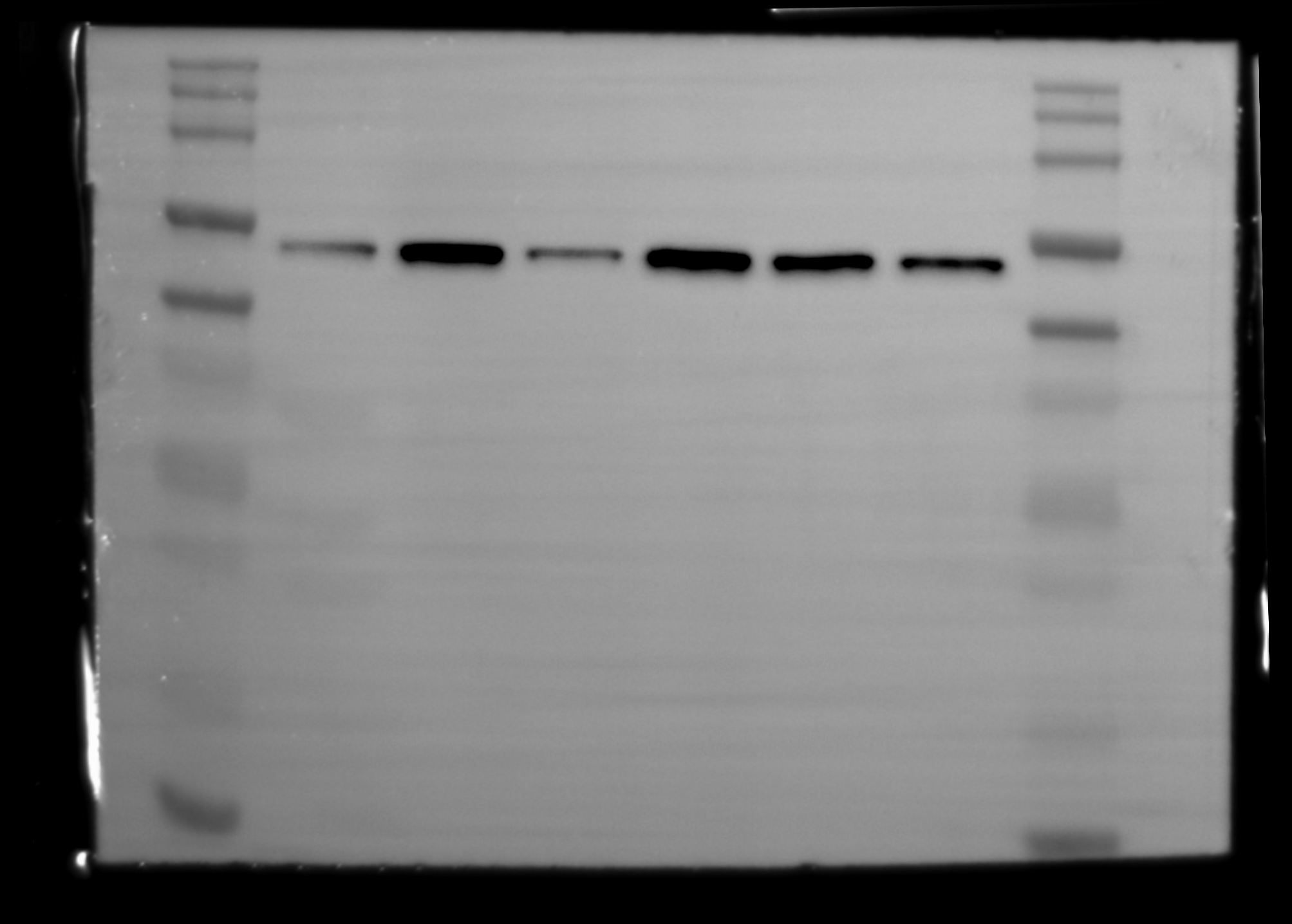


keap1(3)

6.β-actin


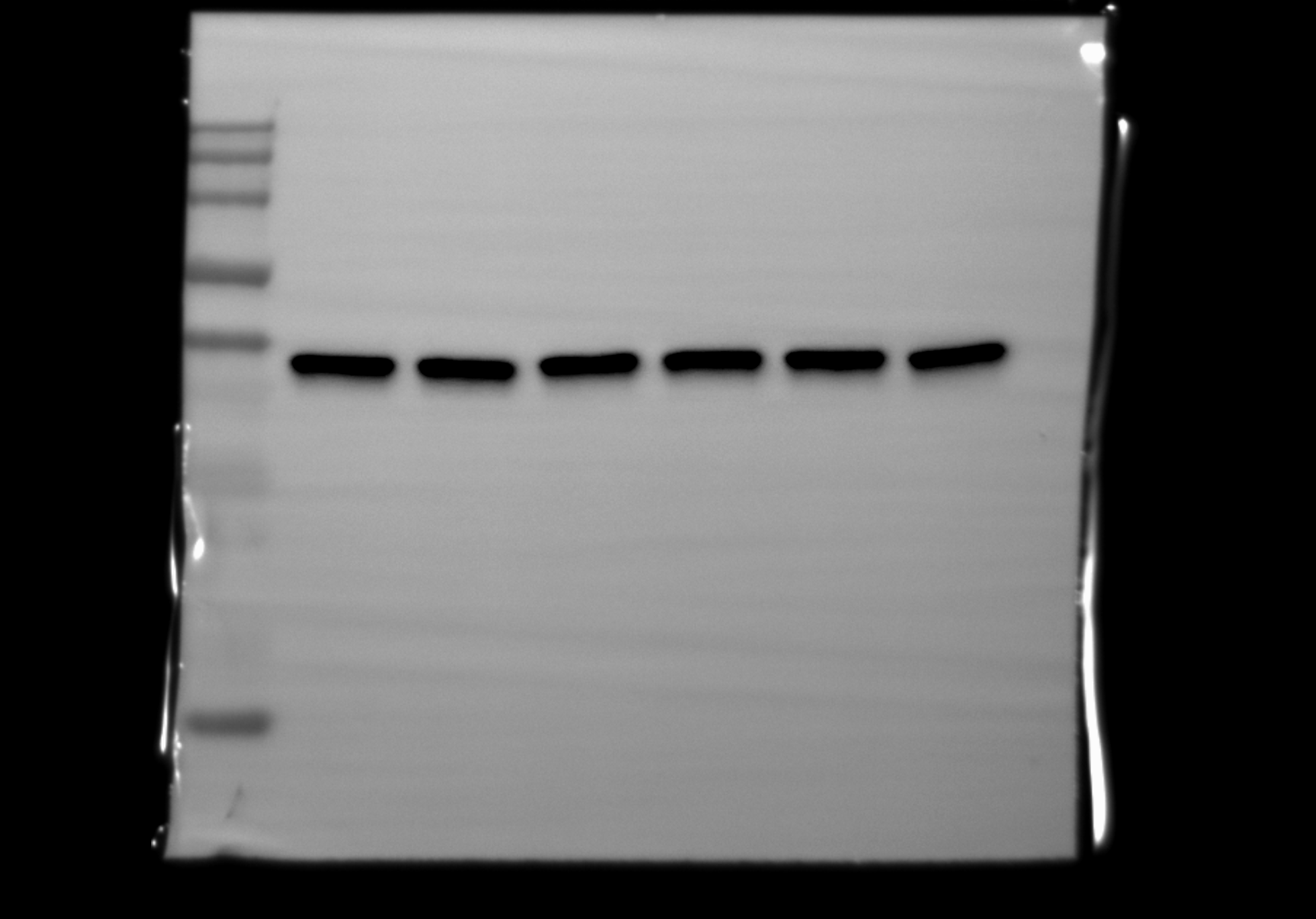

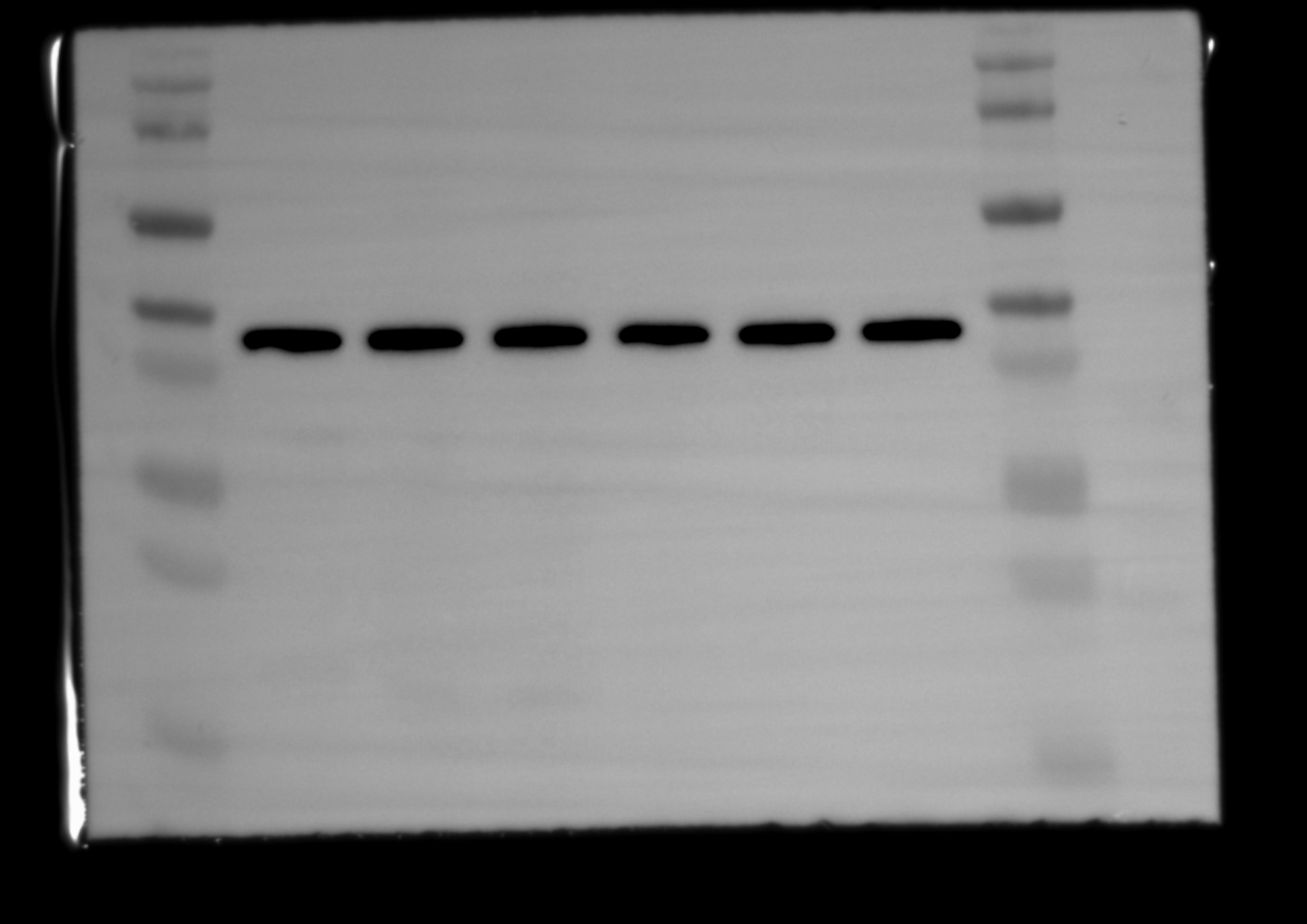


β-actin（1） β-actin（2）

-
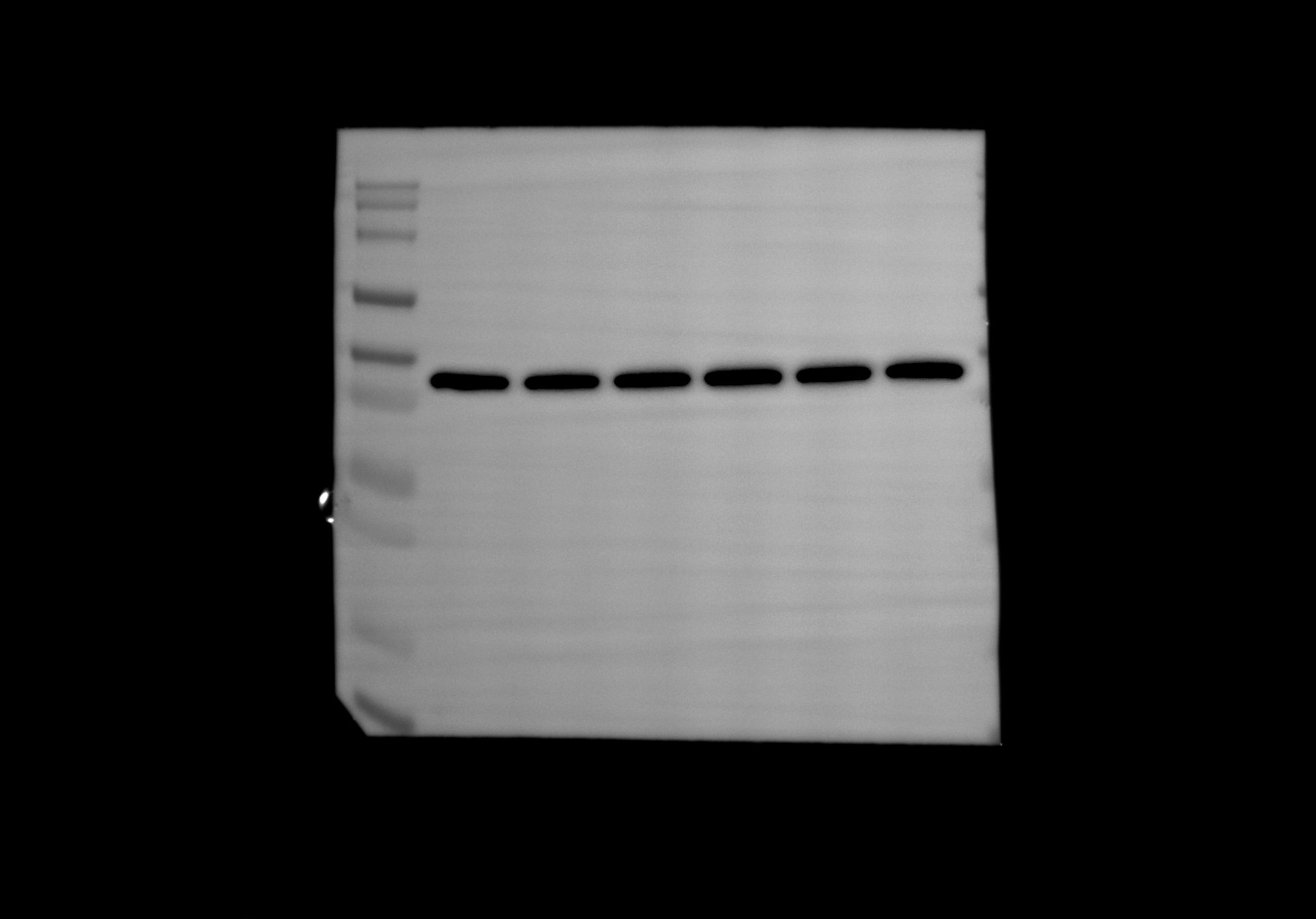

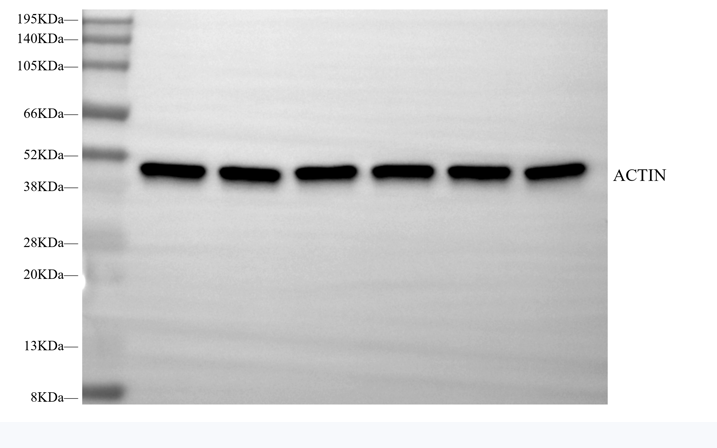


β-actin（3）

Supplement: Supplementary file 6 [file Table4.docx]
